# A bitter melon natural compound ameliorates the myotonic dystrophy type 1 skeletal muscle phenotype in a sex-specific manner

**DOI:** 10.1016/j.neurot.2026.e00978

**Published:** 2026-08-03

**Authors:** Shatha A. Atieh, Chimène Fahmi, Jia Liu, Chunping Tang, Yang Ye, Aymeric Ravel-Chapuis, Bernard J. Jasmin

**Affiliations:** a Department of Cellular and Molecular Medicine, Faculty of Medicine, University of Ottawa, Ottawa, ON, Canada; b Eric J. Poulin Center for Neuromuscular Disease, University of Ottawa, Ottawa, ON, Canada; c China-Serbia “Belt and Road” Joint Laboratory for Natural Products and Drug Discover, Shanghai Institute of Materia Medica, Chinese Academy of Sciences, Shanghai, China; d School of Pharmaceutical Sciences, Faculty of Medicine, University of Ottawa, Ottawa, ON, Canada

**Keywords:** Myotonic dystrophy, Natural compounds, AMPK, Skeletal muscle, Therapeutics

## Abstract

Myotonic Dystrophy Type 1 (DM1) is a multisystemic neuromuscular disease characterized by severe skeletal muscle dysfunction. The etiology of DM1 is primarily driven by RNA toxicity resulting from a gain-of-function mutation in DMPK mRNAs. Beyond this hallmark, DM1 is also characterized by the repression of the AMP-activated protein kinase (AMPK) pathway. Previous work has shown that targeting AMPK represents a novel therapeutic avenue for DM1. In this study, we investigated the therapeutic potential of novel AMPK activators derived from *Momordica charantia* (bitter melon). A screen of 26 bitter melon-derived compounds (BMCs) in C2C12 myotubes identified BMC-25 as a potent AMPK activator. Acute treatment of DM1 (HSA^LR^) mice with BMC-25 induced an expected activation of AMPK in DM1 mice, while chronic treatment restored several DM1 histopathological features, including toxic ribonuclear foci. Interestingly, BMC-25 treatment induced distinct, sex-dependent molecular benefits. In female DM1 mice, BMC-25 treatment corrected the pattern of expression of RNA-binding proteins including CELF1, MBNL1, and Staufen1 in skeletal muscle and achieved a much greater correction of alternative splicing of multiple transcripts relative to their respective controls. In contrast, male DM1 mice exhibited very limited improvements in these parameters. Collectively, our findings indicate that sustained AMPK activation with BMC-25 confers multifaceted benefits to DM1 skeletal muscle by improving core DM1 pathogenic features in a sex-dependent manner. Finally, these results highlight the potential of natural compounds like BMCs as novel, promising and accessible therapeutics for the DM1 muscle pathology.

## Introduction

Myotonic Dystrophy Type 1 (DM1) is an autosomal dominant disease affecting multiple organs and tissues, and is characterized by severe skeletal muscle perturbations [1,2]. The etiology of DM1 is attributed to a gain of function mutation elicited by a CUG repeat expansion (CUG^exp^) in the 3’ untranslated region (UTR) of *DMPK* mRNAs. This, in turn, facilitates the creation of a stable hairpin loop structure in the mutant transcripts, causing their aggregation into toxic ribonuclear foci [[3], [4], [5], [6]]. The number of CUG repeats corresponds to disease severity, with individuals above 50 repeats displaying DM1 symptoms [7]. These toxic foci induce the misregulation and mislocalization of key RNA-binding proteins (RBPs) which cause a multitude of molecular, cellular and histopathological defects. This RNA-mediated mechanism has been referred to as RNA toxicity and is the most widely accepted pathomechanism used to explain the complex disease phenotype [3,[8], [9], [10]]. Among the dysregulated RBPs, Muscleblind-Like Splicing Regulator 1 (MBNL1), CUGBP Elav-Like Family Member (CELF1) and Staufen1 [[11], [12], [13], [14]] markedly contribute to the disease phenotype by promoting a pathological shift in alternative splicing of target mRNAs. As a result, this shift induces a switch from adult to fetal protein isoform expression. This broad missplicing results in the consequent manifestation of the multisystemic DM1 phenotype [[13], [14], [15]]. In skeletal muscle for example, aberrant splicing of *ATP2A1*, *BIN1, CLCN1,* and *RYR1* are strongly linked to key DM1 impairments, including muscle degeneration, myotonia, and muscle weakness.

In addition to the well characterized molecular defects seen in DM1, many signaling pathways have also been shown to contribute to the disease pathogenesis [16]. Key disrupted pathways involved in DM1 skeletal muscle include, protein kinase R (PKR), protein kinase C (PKC), glycogen synthase kinase 3β (GSK-3β), TNF-like weak inducer of apoptosis (TWEAK-Fn14), Akt-mechanistic Target of Rapamycin (Akt-mTOR), Nuclear factor kappa-light-chain-enhancer of activated B cells (NF-kB), calcineurin-nuclear factor of activated T-cell (calcineurin-NFAT), and AMP-activated protein kinase (AMPK) signaling pathways [[17], [18], [19], [20], [21], [22], [23], [24], [25]]. Corrections of different aspects of these pathways either individually or concomitantly have been shown to mitigate key pathological features observed in DM1 [16,20,[26], [27], [28], [29], [30], [31], [32], [33], [34], [35], [36], [37], [38]]. In particular, studies from our lab and others have shown that pharmacological and/or exercise-mediated AMPK activation partially reverses key features associated with the DM1 muscle phenotype; this includes a reduction in the presence of RNA foci, correction in the expression and/or localization of RBPs, and rescue of missplicing events of key transcripts [25]. More specifically, we showed that chronic treatment of DM1 mice with AICAR or MK-8722, both known as AMPK activators, ameliorates these molecular defects in skeletal muscle while also improving key muscle histological features such as central nucleation and cross-sectional area [25,39,40]. Of relevance, the AMPK activator metformin, improves glycemia, mobility and gait of DM1 patients, but had limited effects on splicing even at high doses in DM1 mice [22,41]. Although AICAR and MK-8722 show significant improvements of characteristic DM1 pathological features, they are not FDA approved due to cytotoxicity, while other AMPK activators like metformin, show variable and modest effects [[41], [42], [43]]. Therefore, identifying novel AMPK activators with strong therapeutic potential for DM1 remains critical.

Over centuries, herbal remedies have been extensively used to treat a diverse range of diseases given their rich composition of bioactive compounds [44]. In this context, bitter melon (BM) or *Momordica Charanti*a, has historically been used to treat hyperglycemia and obesity since the 16th century [45,46]. Numerous studies have shown that BM exerts strong antidiabetic properties in insulin target tissues by increasing the quantity of beta-cells and enhancing glucose tolerance through the translocation of GLUT4 [[47], [48], [49], [50]]. This induction of GLUT4 translocation by certain BM extracts has been attributed to the activation of the AMPK pathway [58]. Additionally, BM also improves the plasma lipid profiles in high-fat fed mice by stimulating fatty acid oxidation, and by lowering cholesterol, low density lipoproteins and triglycerides, thereby substantially reducing adipose tissue in these mice [[51], [52], [53], [54], [55], [56]]. Beyond these metabolic benefits, BM also demonstrates anti-inflammatory, antioxidant, and anticancer properties in various preclinical models [[57], [58], [59], [60], [61], [62], [63]]. These effects are largely attributed to its bioactive components, including flavonoids, momorcharin, momordicin, and triterpenoids, which are among the bitter melon’s most therapeutically potent constituents [64,65]. Such findings highlight the importance of investigating the therapeutic potential of BM extracts in other disease contexts driven by dysregulated signaling pathways and the associated cellular and molecular abnormalities that contribute to their pathology.

The objective of our study is to identify and characterize novel AMPK activators derived from the bitter melon fruit (bitter melon compounds or BMC) and evaluate their therapeutic potential for DM1 skeletal muscle. To this end, we characterized 26 BMC and identified one, BMC25, that robustly activates AMPK in cultured myogenic cells and DM1 skeletal muscle. Long-term treatment of DM1 HSA^LR^ mice (HSA^LR^ mice are known to recapitulate several DM1 pathological features [6]) resulted in therapeutic benefits as evidenced by improvements in multiple DM1 pathological muscle features, with some, in a sex-specific manner. Together, these findings: i) highlight the therapeutic potential of BMC to ameliorate the complex DM1 muscle phenotype through AMPK activation; and ii) emphasize the importance of considering sex-based differences in designing and studying potential therapeutic interventions.

## Materials and Methods

### Ethics approval

All procedures and experiments were approved by the University of Ottawa Animal Care Committee and is in compliance of the standards of the Canadian Council on Animal Care and Ontario Animals for Research Act. Animal housing and care were provided by the University of Ottawa Animal Care and Veterinary Service for the full duration of this study.

### Animal models and compound treatment

Three-to seven-month-old male and female DM1 HSA^LR^ mice line LR20b were used in this study (from Dr. Charles Thornton, University of Rochester, Cat# JAX: 032031, RRID: IMSR_JAX 032031). These DM1 transgenic mice carry 250 CTG repeat expansions in under the control of the HSA promoter [6]. DM1 mice were treated daily for 6 weeks and randomly assigned to either the vehicle, AICAR (TRC-A611700, Canada, Toronto, Canada; 500 mg/kg) or BMC-25 treatment group (BMC-25, Roche/Sigma-Aldrich Canada, Oakville, Canada; 1.67 mg/kg). BMC-25 was resuspended in a cocktail vehicle of 5% ethanol, 15% glycerol, and 80% saline. Mice were provided food and water *ad libitum*. Upon the completion of the study, the mice were sacrificed and several hindlimb skeletal muscles were dissected and either (i) flash frozen in liquid nitrogen for protein and RNA extraction or (ii) coated in Tissue-Plus OCT compound (Scigen Fisher HealthCare, Cat # 0004585-03) and frozen in isopentane surrounded by liquid nitrogen for histological and immunofluorescence purposes. More specifically, the left TA muscles were used for RNA and protein extraction for splicing analysis and western blotting, and the right TA muscles were used for histology.

### Cell culture conditions and compound treatment

C2C12 (ATCC, Cat # CRL-1772) were grown in growth medium containing DMEM, 10% fetal bovine serum (VWR, Cat # 35-077-CV), 100 U/mL of penicillin and 100 μg/mL streptomycin to allow cells to attach, grow, and proliferate. To facilitate myoblast differentiation, the growth medium was replaced with 2% horse serum (VWR, Cat # CA45001-058) without the use of antibiotics. These cells were then allowed to differentiate for 5–6 days before undergoing BMC treatment. In order to lower non-specific AMPK activation and induce cell alignment of the metabolic state amongst the myotubes, a serum-starvation of 2 h in DMEM was conducted on the C2C12 myotubes prior to a 1.5-h treatment of BMC compounds. Next, the wells were aspirated, washed with 1X PBS and lysed with 150 μL of 1X RIPA lysis buffer (Milipore Corp, Cat # 20–188). Thereafter, a BCA quantification was conducted to discern protein concentration prior to performing a Western blot analysis to elucidate AMPK activation. BMCs 1–27 for the *in vitro* work were obtained from our collaborators at the Shanghai Institute of Materia Medica. All BMCs were resuspended in 0.1% DMSO and created at a stock concentration of 10 mM as per colleagues Tan et al. These compounds were then compared to the vehicle, 0.1% DMSO and to 2 mM of AICAR (TRC, Cat # A611700, Canada, Toronto, Canada), the positive control.

### MTT assay

In order to test any cytotoxicity associated with BMCs *in vitro*, an MTT assay was conducted on the top BMC candidates in accordance with Roche/Sigma-Aldrich (Cat # 11 465 007 001). C2C12 myotubes were serum-starved for 2 h in DMEM followed by a BMC treatment of 1.5 h 300 μL of MTT reagent was dissolved in 1X PBS and then added to each well. Next, plates were incubated for 4 h at 37 °C before washing with 1X PBS. These crystals were then dissolved by adding DMSO to each well and incubated at 37 °C for 30 min. The plates were then read at 570 nm and 650 nm using a Gen5 microplate reader to calculate cell viability. First, the absorbances were subtracted from each other and then from the negative control (untreated sample). Next, % cell viability was calculated by dividing each sample by the vehicle.

### Western blotting

Dissected TA muscles from female and male DM1 mice were crushed on dry ice prior to resuspension in urea buffer; this buffer contains 7 M urea, 2 M thiourea, 65 mM CHAPS, 100 mM DTT, 125 mM Tris-HCl pH 6.8 and phosphatase and protease inhibitors (PhosSTOP and cOmplete; Roche, Cat # 4906845001, 4693159001). Protein extraction was conducted by centrifuging muscle samples at 20 000 *g* for 15 min at 4 °C. Supernatants were then collected and stored at −80 °C. Protein quantification was conducted using the CB-X protein Assay kit (G-Bioscience, St. Louise, MO, Cat # 786-12X). 20–30 μg of total protein was used for SDS-PAGE to separate proteins prior to transfer onto 0.2 μm nitrocellulose membranes. To prevent, non-specific binding, membranes were blocked for 1 h with 1X TBS-T (1% Tween-20 and Tris-buffered saline) containing Bovine Serum Albumin (Roche/Sigma-Aldrich, Cat # A7906) prior to overnight incubation with the primary antibodies (Table 1). Thereafter, membranes were washed thrice with 1X TBS-T and incubated with their respective horseradish peroxidase-conjugated secondary antibodies (Jackson Immunoresearch Laboratories/Cedarlane) (Table 1). Next, a series of washes were performed prior to incubation with ECL reagents (Bio-Rad, Cat # 170–5061) to reveal their respective signals. Quantifications were normalized to either β-Actin or GAPDH and discerned with Bio-Rad Image Lab software (Image Lab Software, RRID:SCR_014210SCR_014210).

**Table 1:** Series of antibodies used in this study.

Antibody	Concentration	Supplier
AMPK	WB 1:1000	Cell signaling Technology (CST) Cat# 2532, RRID: AB_330331
Phospho-AMPK	WB: 1000	Cell Signaling Technology Cat# 2535, RRID: AB_331250
CUGBP1-HRP-conjugated (CELF1)	WB 1:500	Santa Cruz Cat# sc-20003, RRID: AB_2292334
MBNL1	WB 1:1000 IF 1:250	Abcam: ab45899
Staufen1	WB 1:1000	Abcam: ab73478
β-Actin	WB: 1:5000	Santa Cruz Cat# sc-47778, RRID: AB_626632
GAPDH	WB: 1:100	Cell Signaling Technology Cat# 8884S
Laminin	If: 1:100	Roche/Sigma-Aldrich Cat# L9393, RRID: AB_477163
Goat anti-rabbit A488	IF 1:200	Invitrogen Cat# A-11034 RRID: AB_2576217
Goat anti-rabbit IgG A647	IF 1:200	Thermo Fisher Scientific Cat# A-21244, RRID: AB_2535812

### Alternative splicing approach and analysis

Total RNA was extracted from homogenized TA skeletal muscle powder from either female or male DM1 mice using Tripure Trizol reagent (Roche/Sigma-Aldrich, Cat # 11667165001). Following the addition of 200 μL of chloroform, samples were vortexed for 15 min to induce phase separation for RNA extraction. Samples were then centrifuged at 16 000 g at 4 °C for 15 min. The RNA obtained from the aqueous phase was purified with a DNase kit (Invitrogen/Thermo Fisher Scientific). Next, reverse transcription was conducted to synthesize cDNA by utilizing 500 ng-1 μg of RNA via the RT Master Mix (abm G592; Thermo Fisher). Thereafter, RT-PCR was then performed using GoTaq Flexi DNA polymerase (Promega, Cat #M8291) utilizing mouse primers for *Atp2a1* exon 22, *Ryr1* exon 70, *Clcn1* exon 7a and *Bin1* exon 11 DM1 alternative splicing events (Table 2). These splicing events were run on either a 5% or 8% acrylamide gels, depending on the primer, stained with GelRed dye (Biotium, Cat # 41003 Freemont, CA, USA) and imaged with Gel Doc XR + Imaging System (Bio-Rad, Hercules, CA, USA). Percentage of correction (% inclusion or % exclusion) of alternative splicing events were calculated by averaging the band intensities of either inclusion or exclusion for each respective mouse using Bio-Rad Image Lab software (Image Lab Software, RRID:SCR_014210SCR_014210). Statical significance was determined by using GraphPad Prism (RRID:SCR_002798SCR_002798). Means plus/minus standard error of the mean (SEM) are presented throughout. The levels of significance were defined as follows: ∗*p* < 0.05, ∗∗p ≤ 0.01, ∗∗∗p ≤ 0.001, and ∗∗∗∗p ≤ 0.0001.

**Table 2:** Primer sequences used for RT-PCR analyses in this study.

Target mRNA	Species	PCR primer sequence
*Atp2a1*	Mouse	F: ATCTTCAAGCTCCGGGCCCT R: CAGCTTTGGCTGAAGATGCA
*Bin1*	Mouse	F: TCAATGATGTCCTGGTCAGC R: GCTCATGGTTCACTCTGATC
*Clcn1*	Mouse	F: GGAATACCTCACACTCAAGGCC R: CACGGAACACAAAGGCACTGAATGT
*Ryr1*	Mouse	F: GACAATAAGAGCAAAATGGC R: CTTGGTGCGTTCCTGATCTG

### Fluorescent *in situ* hybridization

Frozen female and male TA skeletal muscle cross-sections (10 μm) were air dried for 30 min at room temperature prior to its fixation in 3% paraformaldehyde in 1X phosphate-buffered saline for 30 min (PBS) (Wisent Bioproducts, Sain-Jean-Baptiste, QC, Canada). Thereafter, sections were lysed and permeabilized by incubation with 0.5% TritonX-100 (Roche/Sigma-Aldrich, Cat # 93443) for 5 min prior to incubation in 30% formamide in 2X UltraPure SSC Buffer (Invitrogen/Thermo Fisher Scientific, Cat # 15557044, Waltham, MA, USA) for 10 min at room temperature. Next, tissue sections underwent a 2-h incubation with a Cy3-labeled (CAG)5 oligonucleotide probe (1 ng/μl; Cat#: F5001; PNA Bio, Thousand Oaks, CA, USA) diluted in FISH buffer (30% formamide, 2X SSC, 0.02% bovine serum albumin (BSA), 66 μg/ml yeast tRNA, 10% dextran sulfate, 2 mM vanadyl ribonucleoside complex) for 2 h at 37 °C. Afterward, a 30 min wash was performed with 30% formamide in 2X SSC followed by two washes with 1X SSC for 30 min. To allow for co-visualization of the foci with MBNL1 (see Table 1), sections were washed 3 times, 10 min each with 1X PBS at room temperature prior to incubation with the blocking solution (1% goat serum albumin and 1% bovine serum albumin in 1X PBS) for 1 h at room temperature. Subsequently, sections were incubated overnight at 4 °C with a MBNL1 primary antibody (see Table 1). Afterward, sections were washed with 1X PBS for 15 min, 3 times each before incubation with an Alexa fluorophore 488- conjugated goat anti-rabbit in 1X PBS (1:200; Invitrogen Cat# A-11034 RRID: AB_2576217) for 1 h at room temperature. Three, 15 min washes were conducted with 1X PBS. Slides were then air dried and mounted with Vectashield Mounting Medium containing DAPI (MJS Biolynx Inc., Cat # H-1200) followed by the addition of a No #1.5 coverslip.

### Muscle histology: central nucleation, CSA and image analysis

Ten micrometer TA muscle cross-sections were stained with anti-laminin primary antibody (Roche/Sigma-Aldrich Cat# L9393, RRID: AB_477163) (Table 1) and thereafter, imaged with a AxioImager M2 upright microscope at 20X magnification (Zeiss, 20x, 0.80 NA, Air, Plan-Apo (BF, DIC, II, DF). The illumination light-source consists of a X-Cite XYLIS LED source at 430 nm, 475 nm, 635 nm and 735 nm. To analyze central nucleation, three separate regions of interest were selected for every cross section present per slide. Each slide contained a total of three or four cross sections. Six to nine ROIs were taken per sample. These regions of interest were selected randomly for cross-sectional area analysis, and the quantity of centrally located nuclei were counted and expressed relative to the total amount of nuclei. Analysis was conducted with the Myosight ImageJ plugin in Fiji software. Means plus/minus SEM are presented throughout. A Welch’s *t*-test or one-way ANOVA was calculated with GraphPad Prism (RRID:SCR_002798) and used to determine whether the differences between groups were statistically significant. The levels of significance were defined as follows: ∗*p* < 0.05, ∗∗p ≤ 0.01, ∗∗∗p ≤ 0.001, and ∗∗∗∗p ≤ 0.0001.

### Image acquisition and analysis

To determine extent of toxic ribonuclear aggregates, images obtained were visualized on the Zeiss Observer O7. It is a widefield light microscope, equipped with the EC Plan-Neofluar (0.085 NA, air), the 20x, Plan-Apochromat (0.8NA, air), and the 40x, Plan Apochromat (NA1.4, oil), DIC. The illumination light-source consists of LED-Colibri7 with 385 nm, 430 nm, 475 nm, 555 nm, 590 nm, 630 nm, 735 nm LED lines. Images were detected and acquired using the Zeiss Axiocam 705 monochrome camera.

Each slide contained three or four tissues sections. Three random areas of interest were chosen per-cross section and imaged at 20X magnification (0.8 NA, air) using the Z-stack acquisition setting mode to detect nuclei, RNA foci, and MBNL1 RBP. Depending on the cross-section, the acquired Z-stacks consisted of 15–23 focal planes at 0.49 μm intervals. At least 9 images were taken per slide. Next, images were deconvoluted using the Zen 3.10 pro software. Afterward, FISH staining images were analyzed using Imaris (RRID:SCR_007370) and the Imaris Spot (foci present in the nucleus) or ImarisSurface (foci with MBNL1) Detection artificial intelligence algorithms to classify nuclei (DAPI), RNA foci and co-localized foci and MBNL1 RBPs. The number of nuclei colocalized with or without nuclear RNA foci (or with or without foci and MBNL1) was measured using the Imaris software and expressed as a percentage of either the total number of nuclei identified (an average of at least 28–175 nuclei were identified) or total number of foci (or total number of foci with MBNL1). This percentage was obtained by dividing the number of foci-positive nuclei (or the total number of foci containing MBNL1) by the total number of nuclei identified per image. Subsequently, the number of foci-positive nuclei (or the total number of foci containing MBNL1) per cross-section per slide was averaged and attributed to the correct mouse.

### Statistical analysis

A Welch *t*-test or one-way ANOVA was used to performed statistical differences between experimental cohorts. The level of statistical significance was set at a *P*-value <0.05 (∗*P*-value <0.05, ∗∗*P*-value ≤0.01, ∗∗∗*P*-value ≤0.001 and ∗∗∗∗*P*-value ≤0.0001). The error bars correspond to the SEM. The extent of statistical significance was determined using the GraphPad Prism 9 (GraphPad Prism: SCR_002798).

## Results

### BMC-25 activates AMPK and maintains high cell viability in C2C12 myotubes

Our lab previously demonstrated that AMPK activation is markedly repressed in DM1 muscle [25,39,40], and we and others have shown that pharmacological (AICAR or MK-8722) and/or physiological (exercise) activation of AMPK can reverse several pathological features of DM1 [39,40], [25,39,66]. These findings indicate that AMPK activation is an attractive therapeutic target for DM1 and highlights the need to identify novel compounds capable of mitigating core aspects of the DM1 pathology while also allowing for clinical transferability.

**Fig. 1:**
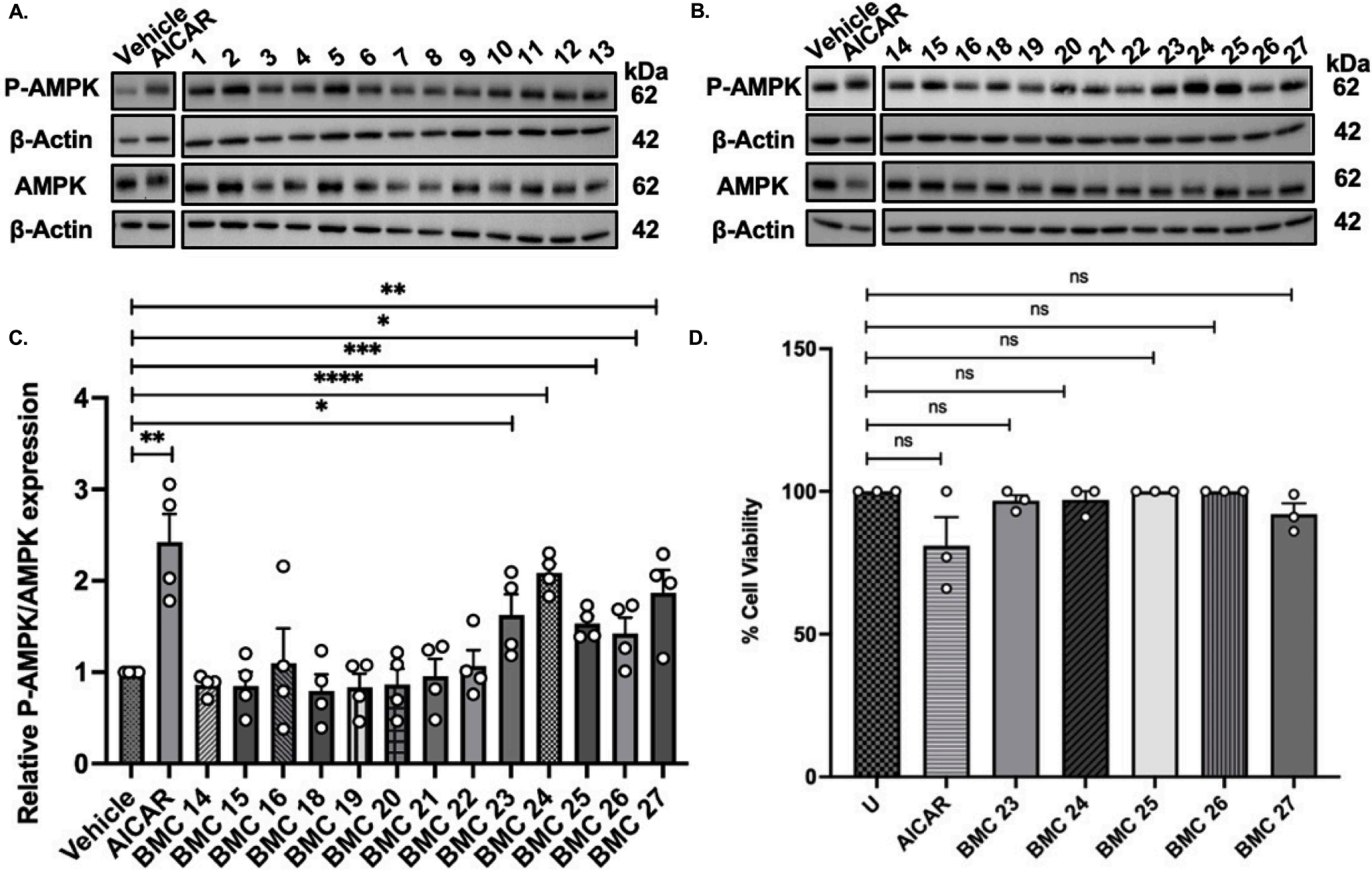
**BMCs activate AMPK while maintaining high cell viability in C2C12 myotubes.** (A, B) Representative Western blots of AMPK activation in C2C12 myotubes after treatment with BMCs or AICAR. (C) Phospho-AMPK was quantified over total AMPK level after β-Actin normalization to discern differences in AMPK activation. *N* = 4. BMCs 23, 24, 25, 26 and 27 activate AMPK similarly to AICAR relative to the vehicle. (D) Best BMCs have a cell viability score to the same degree as the untreated control. *N* = 3. U: untreated control, AICAR: 2 mM of AICAR (positive control), BMC: 10 μM of Bitter melon compound. A Welch’s one-tail *t*-test was performed (ns: *P*-value ≥0.05, ∗*P*-value <0.05, ∗∗*P*-value ≤0.01, ∗∗∗*P* ≤ 0.001, ∗∗∗∗*P* ≤ 0.0001 relative to the vehicle).

To this end, 26 BMCs were examined for their ability to induce phosphorylation of AMPK in cultured C2C12 mouse myotubes via Western blotting. A 2 mM concentration of AICAR, used as a positive control, was chosen in accordance with the experiments published by our lab and others [25,39,56,66]. To lower non-specific AMPK activation and induce cell alignment of the metabolic state amongst C2C12 myotubes, a serum-starvation of 2 h in DMEM was conducted (see Methods above) prior to a 1.5-h treatment with the various BMCs. Of these compounds, # 23, 24, 25, 26 and 27 were identified to significantly induce AMPK activation in C2C12 myotubes in various degrees. Compared to the vehicle, AICAR caused a ∼2.4-fold activation of AMPK (*P* < 0.01), while BMCs 23, 24, 25, 26 and 27 resulted in ∼ a 1.5-to-2.1-fold increase in AMPK (*P* < 0.05, *P* < 0.0001, *P* < 0.001, *P* < 0.05, *P* < 0.01) (Fig. 1B and C). These extracts were selected as the top candidates (Fig. 1). Results obtained with BMC #1 to 13 did not significantly increase AMPK phosphorylation (Fig. 1A).

Next, MTT assays were conducted with the top BMCs to assess *in vitro* cytotoxicity. This assay was performed in accordance with the protocol published by Roche/Sigma-Aldrich (see Methods). Our results show that the top BMCs maintain cell viability by ∼92%, while AICAR resulted in 86% cell viability (Fig. 1D).

### Activation of AMPK by BMC-25 in DM1 mice

**Fig. 2:**
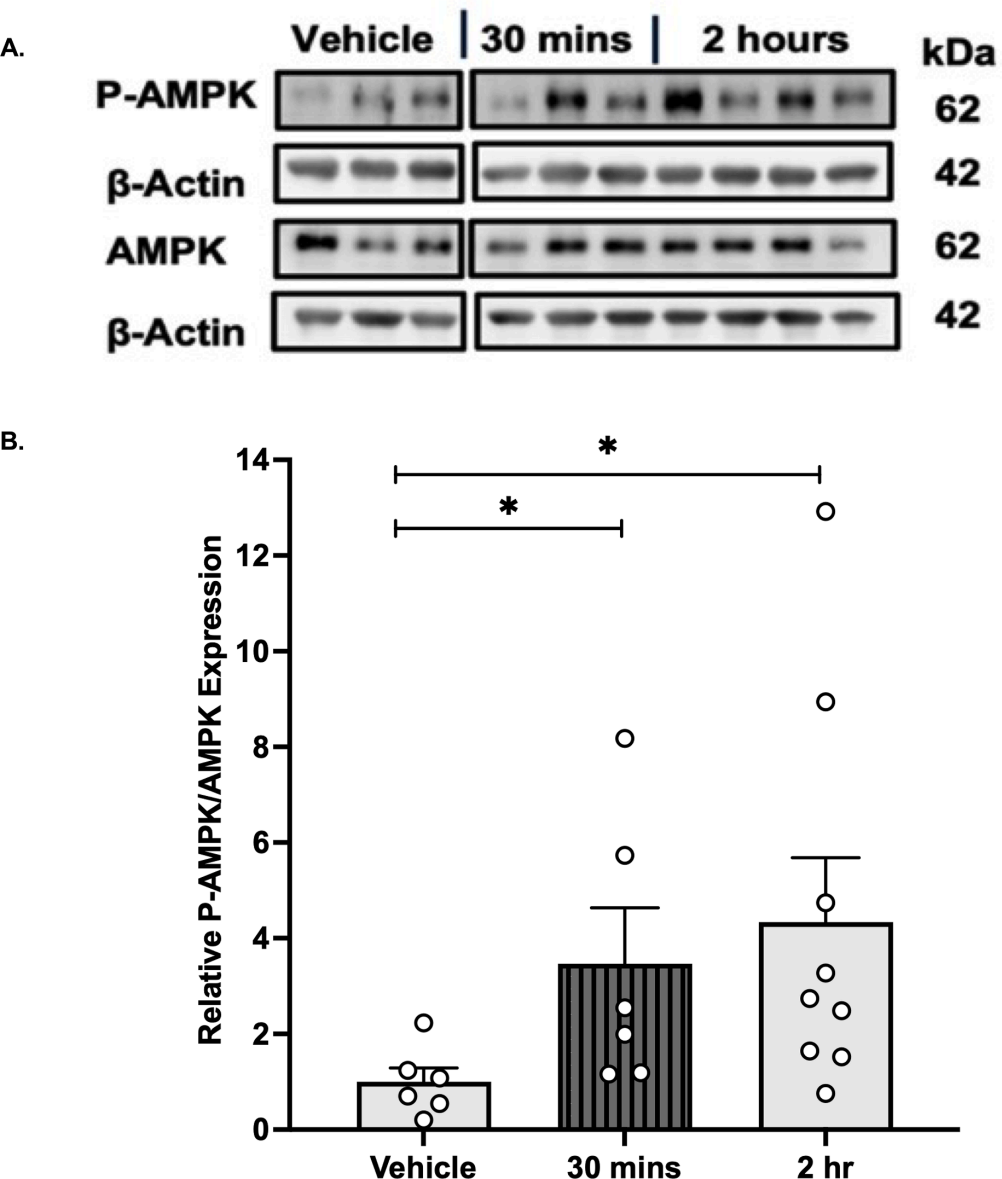
**BMC-25 shows a strong activation of AMPK at 30 min and 2 h in DM1 mice. (A, B) Western blots and quantification of Phospho-AMPK and AMPK in TA muscles**. (A) Expression levels of phospho-AMPK (P-AMPK) and total AMPK (AMPK) to β-Actin from the TA of 3–7 -month-old (HSA^LR^) DM1 mice. (B) Phospho-AMPK was quantified over total AMPK to discern differences in AMPK activation in DM1 mice with mean ± SEM. *N* = 6–9 mice. AICAR: 500 mg/kg of AICAR, positive control, BMC-25: 1.67 mg/kg of Bitter melon compound. A Welch’s one-tail *t*-test was performed (∗*P* < 0.05 relative to the vehicle).

Due to the extensive investigation of BMC-25 in other disease contexts and its marked induction of AMPK in preclinical models [56,67], we chose this compound for further investigation in the DM1 mice [[55], [56], [57], [58], [59], [60], [61], [62], [63], [64]]. In order to determine the pattern of AMPK activation in DM1 skeletal muscle, three-to seven-month-old DM1 (HSA^LR^) mice were treated with BMC-25 for 30 min and 2 h (Fig. 2). Western blots were then performed to determine the extent of AMPK phosphorylation using the tibialis anterior (TA) muscles of these mice (Fig. 2). Our findings show that BMC-25 activates AMPK levels (Thr172) in TA muscles of DM1 mice after 30 min and 2 h of treatment relative to vehicle-treated mice (*P* < 0.05). Such findings indicate that BMC-25 is a strong candidate for further in-depth investigation of its therapeutic efficacy in DM1.

### Improved muscle fiber morphology following BMC-25 treatment in DM1 mice

**Fig. 3:**
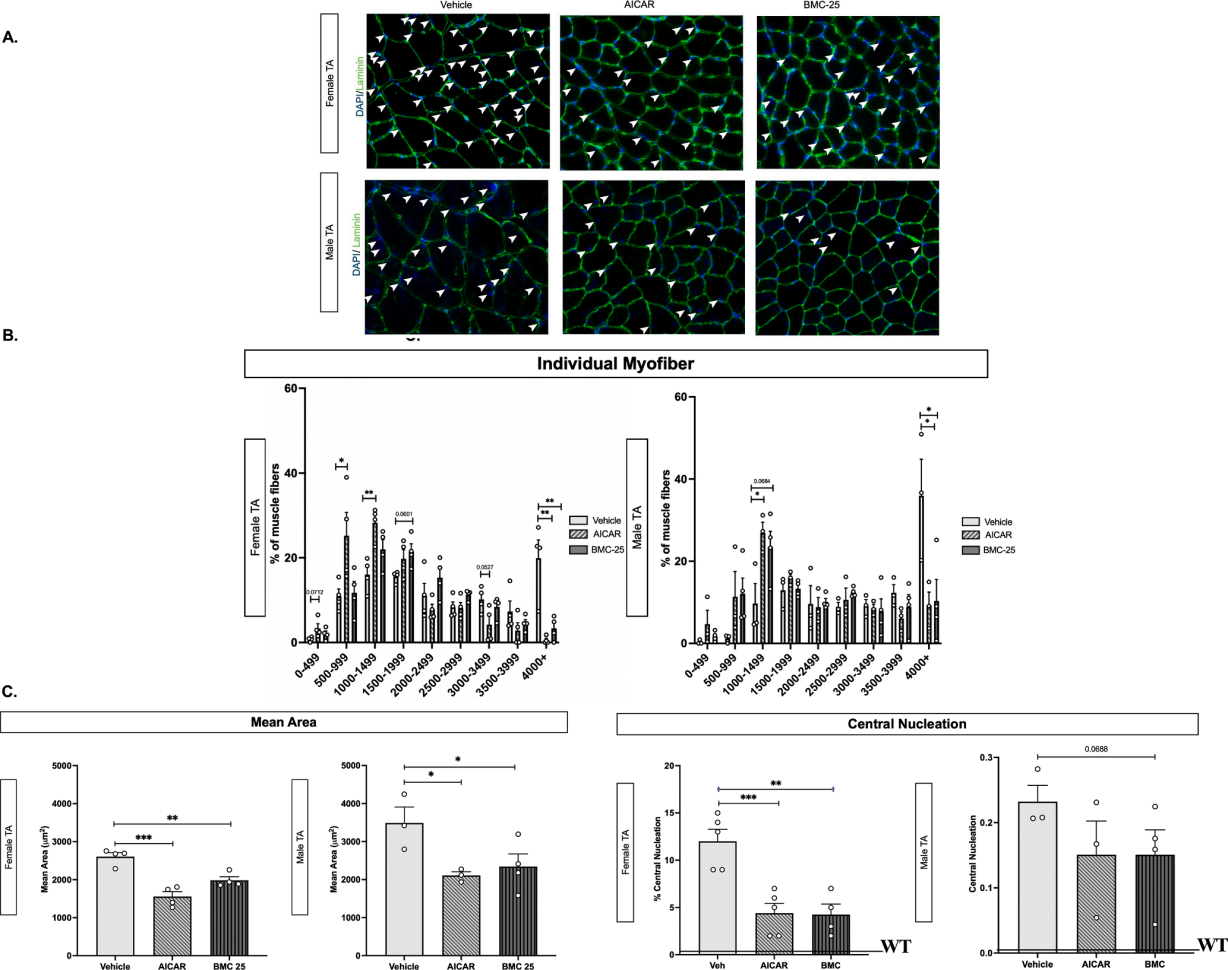
**Six weeks of BMC-25 administration in DM1 mice ameliorates histopathological features in TA muscle.** (A) Representative laminin staining of TA muscles. Central myonuclei are indicated by arrows. ( (B) Individual and (C) average myofiber cross-sectional area (CSA) are shown in the TA of 3-7-month-old DM1 (HSA^LR^) mice (D) Percentage of central nucleation after BMC-25 or AICAR treatmnet in DM1 mice with mean ± SEM. Wild-type (WT) data is represented by the horizontal line on the graphs in panel D. *N* = 7–8. AICAR: 500 mg/kg of AICAR, positive control, BMC-25: 1.67 mg/kg of Bitter melon compound. A one-way ANOVA was conducted on B, and a Welch’s one-tail *t*-test was performed on C and D(∗*P*-value <0.05, ∗∗*P*-value ≤0.01, ∗∗∗*P* ≤ 0.001 relative to the vehicle). Scale bars, 50 μm.

DM1 skeletal muscle fibers display several abnormalities including an increase in central nuclei which is indicative of enhanced muscle regeneration [68]. Accordingly, we first examined whether chronic treatment of DM1 mice with BMC-25 would reduce these DM1 histopathological defects. Male and female DM1 mice were randomly assorted into one of three cohorts: vehicle, AICAR or BMC-25-treated groups. Mice in the treatment group received daily subcutaneous injections of either AICAR or BMC-25 for 6 weeks while mice in the control group received the vehicle also daily for 6 weeks. Afterward, mice were euthanized, and immunofluorescent staining was performed on cross-sections of their TA muscle with an anti-laminin primary antibody (Table 1) and 4’,6-diamidino-2-phenylindole (DAPI). This allowed for the visualization of nuclei while also defining the periphery of individual muscle fibers. Our previous work has also shown that the CSA of DM1 mice is typically shifted toward larger, more hypertrophic muscle fibers (>4000 ${\;\mu m}^{2}$) in DM1 mice [25,39,40]. An analysis of the distribution of individual CSA of TA muscles from treated DM1 mice indicated that both long-term treatments, i.e, AICAR and BMC-25 significantly (*P* < 0.01 and *P* < 0.05, respectively) reduced the relative number of larger muscle fibers (>4000 ${\;\mu m}^{2}$) in DM1 mice relative to vehicle in both female (*P* < 0.01) and male (*P* < 0.05) DM1 mice (Fig. 3B). Additionally, the 6-week treatment with the AMPK activator AICAR promoted a shift of fiber size distribution that more closely resembles wild-type (WT) values. This was more significant in the female cohort than in their male DM1 counterpart (see previous reports [25,39,40]) (Fig. 3B). This adaptation includes a significant increase of fibers with smaller CSAs following BMC-25 treatment (Fig. 3B).

We also measured the average CSA of muscle fibers following long-term treatments in DM1 mice (Fig. 3C). As expected, our results show significant reductions in the average CSA following AICAR (*P* < 0.001) or BMC-25 (*P* < 0.01) treatments in female DM1 mice as well as in male DM1 mice (AICAR (*P* < 0.05) and BMC-25 (*P* < 0.05)) (Fig. 3C). Histological improvements were thus consistent across both sexes with regards to these parameters. However, to clearly distinguish the effects of BMC-25 treatment between the female and male DM1 cohorts, their respective datasets were separated for analysis. Altogether, these data highlight the ability of BMC-25 to restore several morphological features of DM1 muscle fibers.

Finally, the number of central nuclei of treated muscle fibers was then determined. As illustrated in Fig. 3, the quantity of centrally located nuclei was significantly ((*P* < 0.001) reduced in the TA muscles of DM1 mice following daily injections of AICAR for 6 weeks in only the female DM1 mice, as expected and based on our previous work [25,39,40] (Fig. 3A,D). Similarly, long-term treatment of DM1 mice with BMC-25 also reduced (*P* < 0.01) the number of central nuclei relative to their vehicle counterparts in the female cohort (Fig. 3A,D). AICAR and BMC-25 treatment only showed a trend toward reductions in central nucleation in the male DM1 mice (Fig. 3A,D).

### BMC-25 reduces nuclear aggregation of toxic CUG^exp^ mRNAs

**Fig. 4:**
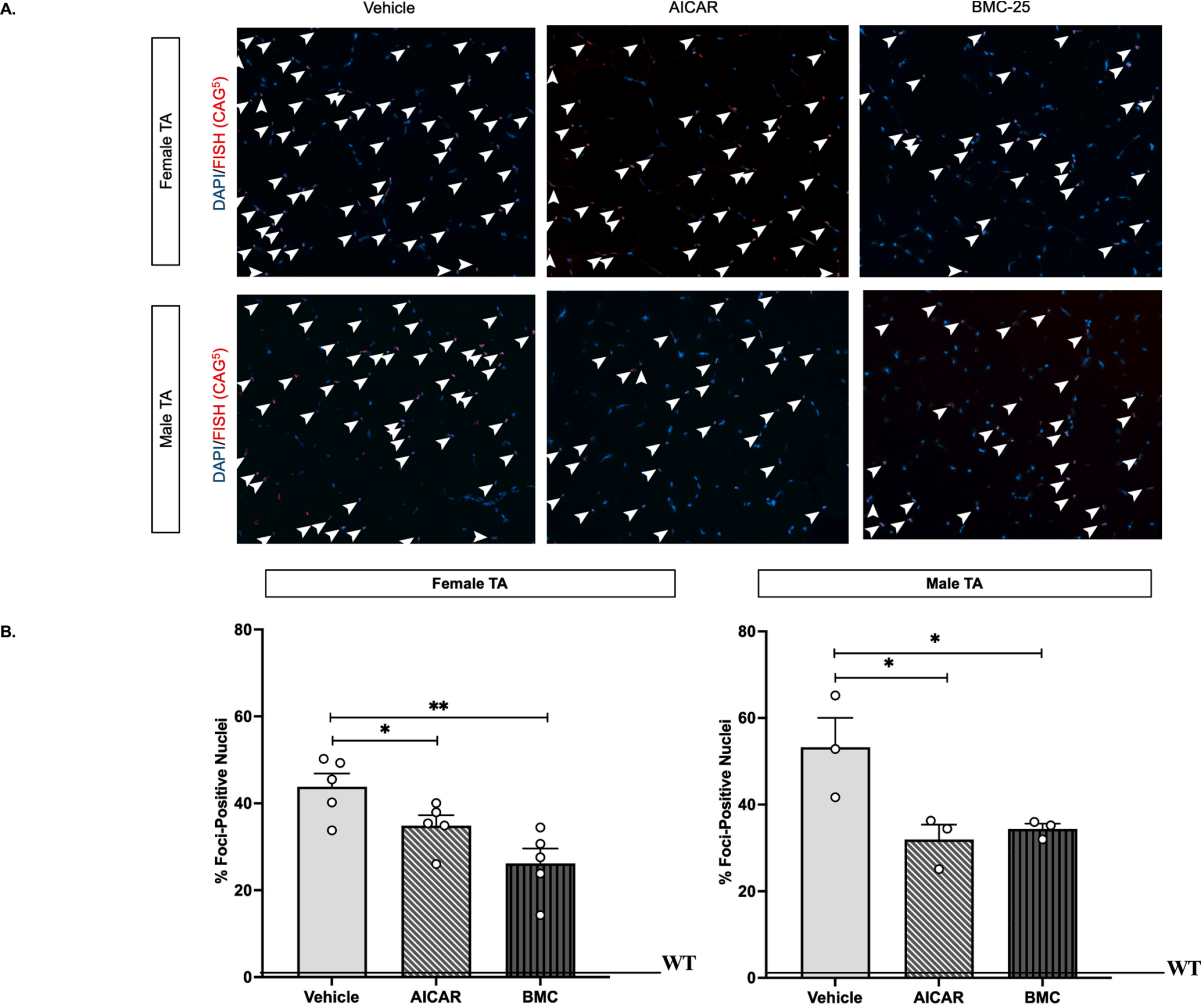
**Chronic treatment of BMC-25 in DM1 mice reduces toxic nuclear foci in their TA muscles**. (A) Representative FISH staining of TA muscles of 3-7-month-old female and male DM1 (HSA^LR^) mice. Colocalization of nuclei (blue) and CUG^exp^ RNA foci (red) are indicated by arrows. (B) Percentages of RNA-foci positive nuclei after BMC-25 or AICAR treatment in the TA of female and male mice are shown with mean ± SEM. Wild-type (WT) data is represented by the horizontal line on the graphs in panel B. *N* = 8. AICAR: 500 mg/kg of AICAR, positive control, BMC-25: 1.67 mg/kg of Bitter melon compound. A Welch’s one-tail *t*-test was performed, (∗*P*-value <0.05, ∗∗*P*-value ≤0.01 relative to the vehicle). Scale bars, 50 μm.

**Fig. 5:**
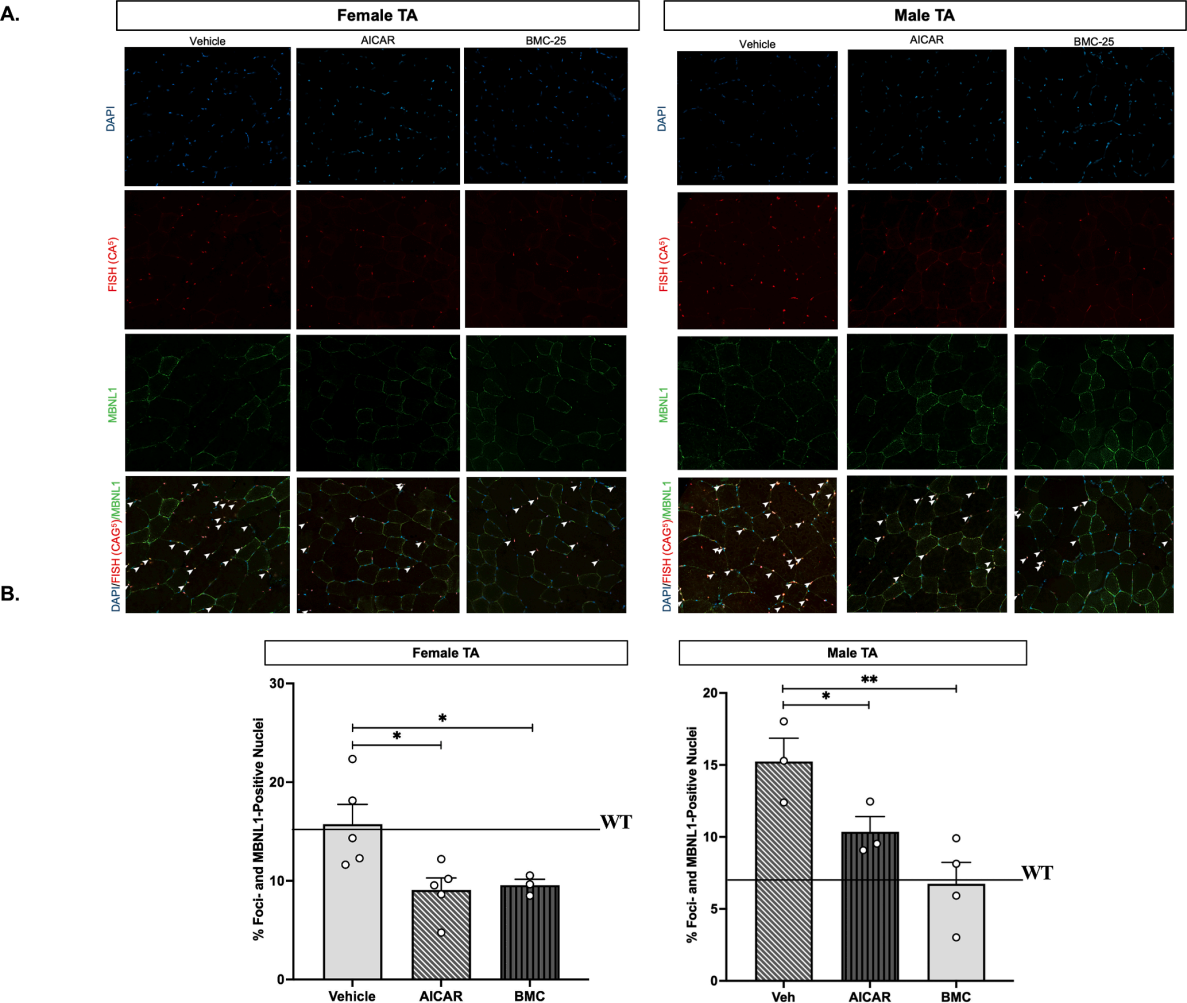
**Treatment with BMC-25 for six weeks decreases the colocalization of nuclear foci with MBNL1 in the TA muscles of DM1 mice**. (A) Representative FISH and IF staining of TA muscles of 3-7-month-old female and male DM1 (HSA^LR^) mice . Colocalization of nuclei (blue), CUG^exp^ RNA foci (red) and MBNL1 (green) are indicated by arrows. (B) Percentages of RNA foci -and MBNL1-positive nuclei after BMC-25 or AICAR treatment in the TA of female and male DM1mice are shown with mean ± SEM. Wild-type (WT) data is represented by the horizontal line on the graphs in panel B. *N* = 7–8. AICAR: 500 mg/kg of AICAR, positive control, BMC-25: 1.67 mg/kg of Bitter melon compound. A Welch’s one-tail *t*-test was performed (∗*P*-value <0.05,∗∗*P*-value ≤0.01 relative to the vehicle). Scale bars, 50 μm.

Pathogenic features of DM1 arise from a toxic gain-of-function elicited by expanded CUG repeats in *DMPK* mRNAs, resulting in the formation of nuclear RNA foci that disrupt the expression and/or localization of key RBPs, including MBNL1 [12,13]. To investigate the potential effect of BMC-25 treatment on reducing toxic RNA foci, we conducted fluorescent *in situ* hybridization (FISH) analyses on TA skeletal muscles of DM1 mice. TA cross-sections from mice in each treated group were incubated with a (CAG)5 Cy3-labeled oligonucleotide probe to visualize toxic RNA foci. Nuclei in these cross-sections were visualized by co-staining with DAPI. We then determined the extent of CUG^exp^ foci-positive nuclei as well as MBNL1 sequestration by colocalizing each respective fluorescent signal. As we previously observed, our findings here show that AICAR-treatment of DM1 mice resulted in a significant correction in the number of toxic RNA foci (*P* < 0.05) in both female and male DM1 mice and in MBNL1 expression (*P* < 0.05) after long-term treatment compared to vehicle and WT cohorts [25,39,40] (Fig. 4, Fig. 5). Similarly, BMC-25 treatment also resulted in a significant reversal of nuclear foci accumulation in both female (*P* < 0.01) and male (*P* < 0.05) DM1 mice. Moreover, BMC-25 treatment led to a significant reduction in and MBNL1 sequestration in female (*P* < 0.05) and male (*P* < 0.01) DM1 mice relative to their vehicle-treated and WT [25,39,40] counterparts (Fig. 4, Fig. 5).

### Correction of misregulated RNA-binding protein expression by BMC-25 treatment

**Fig. 6:**
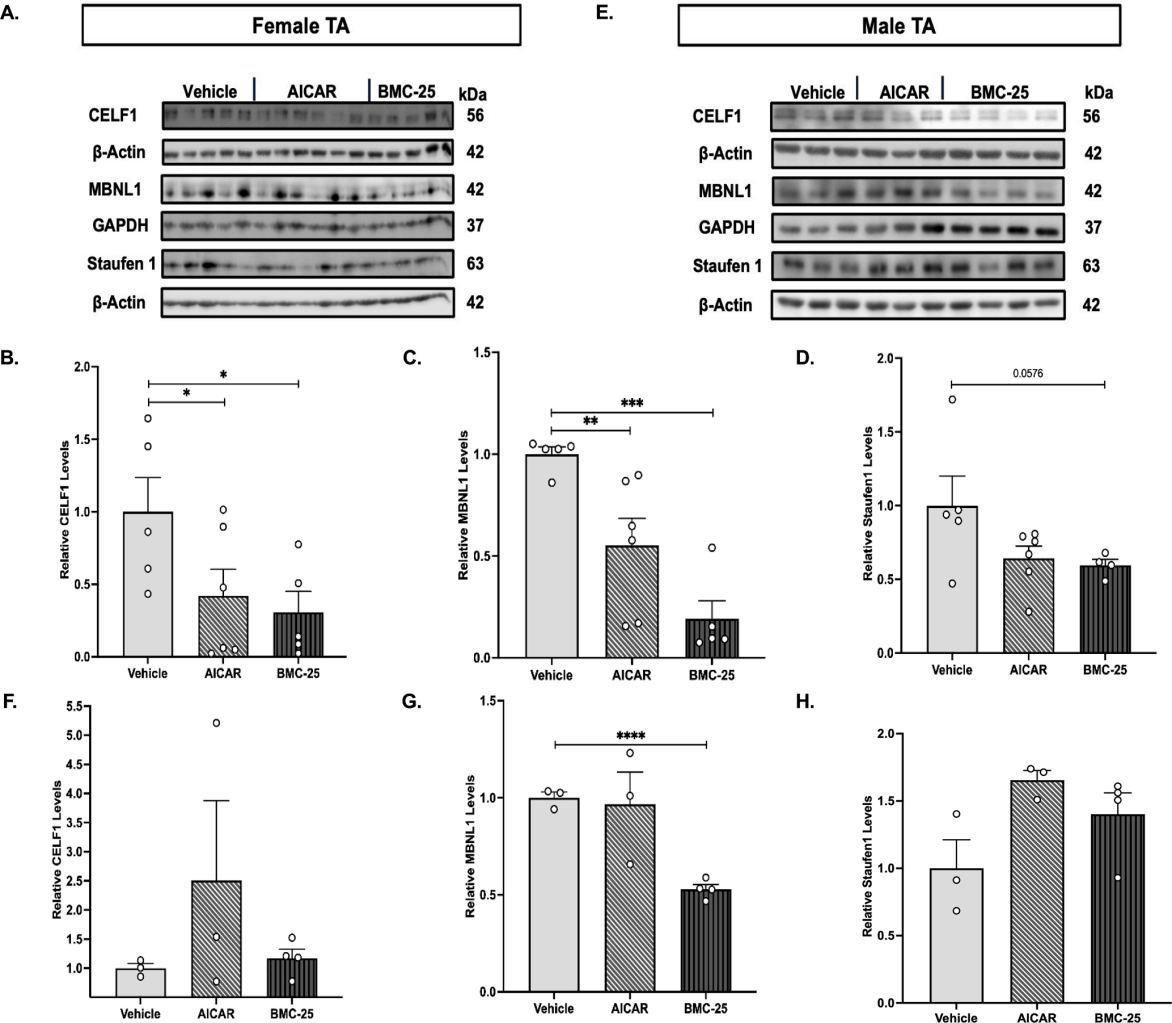
**Prolonged treatment of BMC-25 in DM1 mice results in reduction of CELF1, MBNL1 and Staufen1 in TA muscles of Female DM1 mice**. Normalized expression levels of CELF1, MBNL1 and Staufen1 with their representative western blots relative to β-Actin or GAPDH from the TA of female (A) or male (E) of 3–7 -month-old DM1 (HSA^LR^) mice. CELF1, MBNL1 and Staufen1 were quantified over β-Actin or GAPDH to discern differences in their relative expression levels in female (B–D) and male (F–H) DM1 mice with mean ± SEM. *N* = 3–6 mice. AICAR: 500 mg/kg of AICAR, positive control, BMC-25: 1.67 mg/kg of Bitter melon compound. A Welch’s one-tail *t*-test was performed (∗*P*-value <0.05, ∗∗*P*-value ≤0.01, ∗∗∗*P*-value ≤0.001 and ∗∗∗∗*P*-value ≤0.0001 relative to the vehicle).

In DM1, toxic RNA aggregates lead to a loss-of-function of MBNL1 and a gain-of-function of CELF1 and Staufen1, thereby driving the DM1 spliceopathy. In the current study, we analyzed the expression of CELF1, MBNL1, and Staufen1 using TA muscle extracts from AICAR and BMC-25 treated DM1 mice. Interestingly, and in contrast to the AMPK activation and morphological data shown above (Fig. 2, Fig. 3, Fig. 4, Fig. 5), our results revealed a sex-specific difference in the levels of these RBPs following treatment with AICAR or BMC-25. More specifically, CELF1 (*P* < 0.05) and MBNL1 (*P* < 0.001) protein levels were significantly reduced in TA muscles of female DM1 mice treated with BMC-25 (Fig. 6A–C). A strong similar trend (*P* = 0.0576) was also observed with Staufen1 in BMC-25-treated female DM1 mice (Fig. 6D). Chronic AICAR treatment also led to reductions of some RBPs in female DM1 mice (Fig. 6A–C). By contrast, only MBNL1 was significantly decreased (*P* < 0.0001) by BMC-25 treatment with no effect (P ≥ 0.05) on the expression of CELF1 and Staufen1 in TA muscles from male DM1 mice (Fig. 6E–H). Furthermore, AICAR administration failed to affect the levels of these RBPs (Fig. 6E–H). Taken together, these results indicate a sex-dependent response of RBPs following treatment of DM1 mice with either AICAR or BMC-25.

### BMC-25 treatment preferentially rescues mis-splicing of key RNA targets in female DM1 mice

**Fig. 7:**
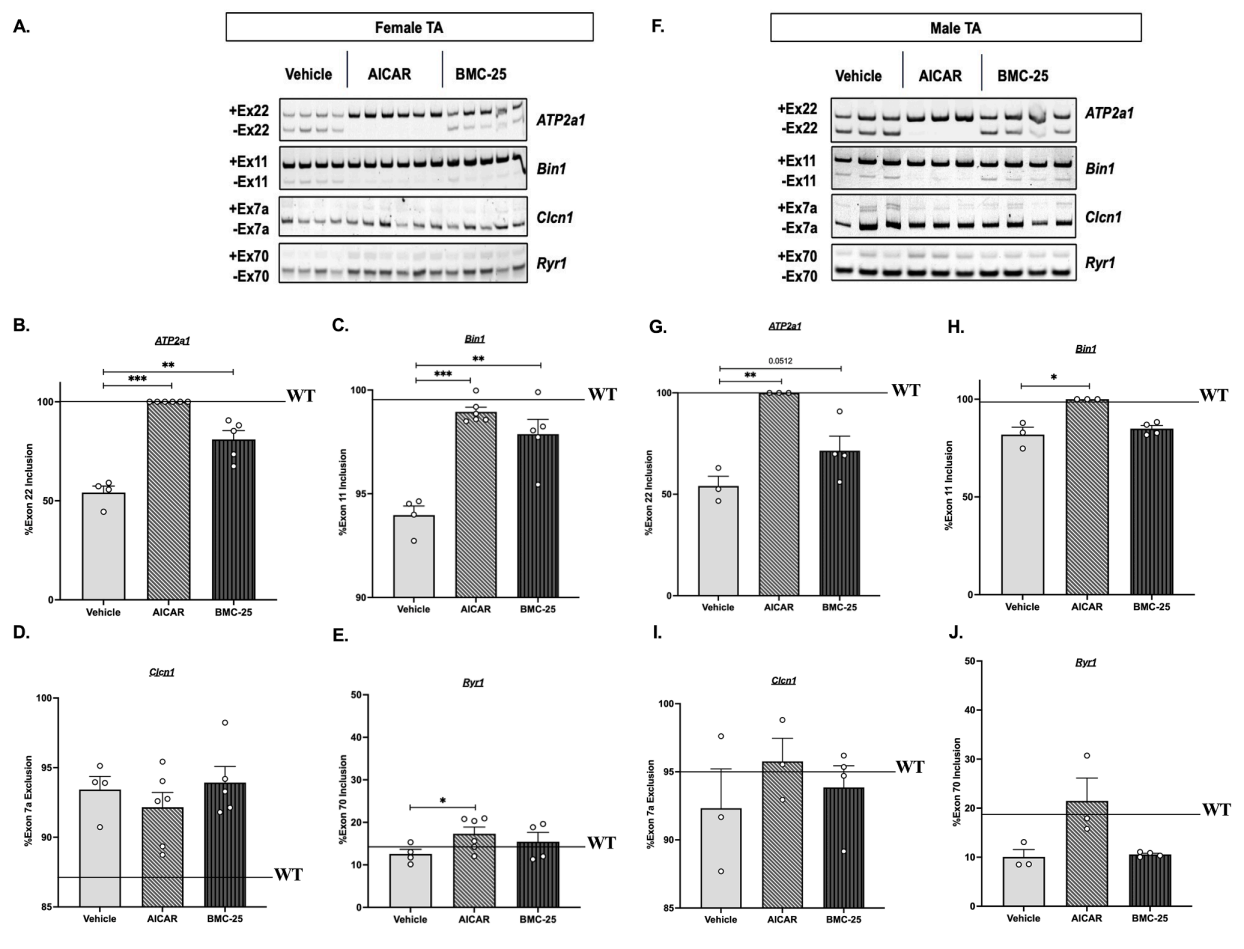
**Long-term treatment of BMC-25 in female and male DM1 mice corrected key misspliced transcripts in the TA muscles of the female cohort**. Representative RT-PCR analysis showing the splicing patterns of *Atp2a1* exon22, *Bin1* exon11, *Clcn1* exon7a and *Ryr1* exon70 in the TA of BMC-25- and AICAR-treated 3–7 -month-old female (A) and male (F) DM1 (HSA^LR^) mice. Percentages of (B, G) *Atp2a1* exon22 inclusion, (C, H) *Bin1* exon11 inclusion, (D, I) *Clcn1* exon7a exclusion, and (E, J) *Ryr1* exon70 inclusion are shown with mean ± SEM in female (B–E) and male (G–J) DM1 mice. Wild-type (WT) data is represented by the horizontal line on the graphs in panels B-E and G-J. *N* = 4–6. AICAR: 500 mg/kg of AICAR, positive control, BMC-25: 1.67 mg/kg of Bitter melon compound. (A Welch’s one-tail *t*-test was performed ∗*P*-value <0.05, ∗∗*P*-value ≤0.01, ∗∗∗*P* ≤ 0.001 relative to the vehicle).

The misregulation and mislocalization of RBPs in DM1 cause aberrant splicing events [13,14], which underlie symptoms such as myotonia and skeletal muscle weakness [14]. Given the observed differences in the therapeutic response of RBP levels following BMC-25 or AICAR treatments (see Fig. 6), and our prior findings showing sex-specific differences in alternative splicing in DM1 mice following treatment with either AICAR, AICAR plus exercise, or with MK-8772 [40], we examined whether long-term BMC-25 treatment could rescue key misspliced DM1 transcripts in muscles from male and female DM1 mice using RT-PCR and gel electrophoresis. In our analyses, we included splicing patterns of *Atp2a1* exon 22 [69], *Bin1* exon 11 [70], *Ryr1* exon 70 [71], and *Clcn1* exon 7a [72]. Baseline values for the wild-type (WT) cohort were sourced from previously published data by Misquitta et al. (2024) [39]. Similar to our previous work [39,40], TA muscles from AICAR-treated female DM1 mice showed a correction of *Atp2a1* exon 22 (*P* < 0.001), *Bin1* exon 11 (*P* < 0.001), and *RyR1* exon 70 (*P* < 0.05) with no change in the splicing pattern of *Clcn1* exon 7a (Fig. 7A–E). Similarly, BMC-25 treatment in female DM1 mice corrected (*P* < 0.01) the missplicing of *Atp2a1* exon 22 and *Bin1* exon 1 (Fig. 7A–E).

In male DM1 mice, 6-weeks of daily subcutaneous injections with AICAR resulted in similar results as seen in our previous work [39,40]. In particular, there was a correction of *Atp2a1* exon 22 (*P* < 0.01), and *Bin1* exon 11 (*P* < 0.05) splicing with no significant improvement of *Clcn1* exon 7a or *Ryr1* exon 70 in TA muscles from these mice (Fig. 7). Moreover, long-term treatment with BMC-25 resulted only in a trend toward correction (*P* = 0.0512) of *Atp2a1* exon 22 (Fig. 7F, G) with no significant correction of *Bin1* exon 11, *Clcn1* exon 7a or *Ryr1* exon 70 in the TA muscles of male DM1 mice (Fig. 7F–J).

**Fig. 8:**
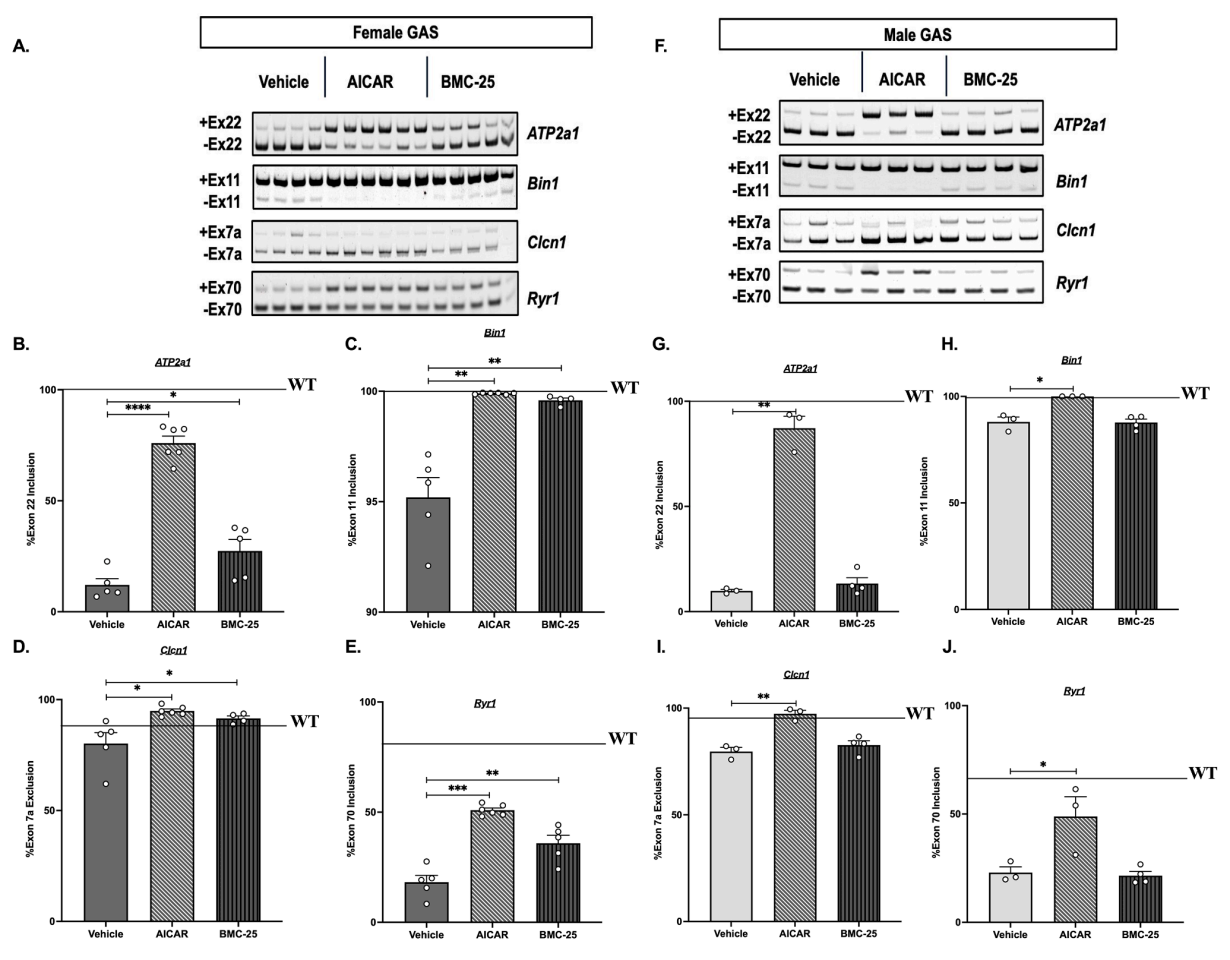
**Long-term treatment of BMC-25 in female and male DM1 mice showed a sex-specific rescue of misspliced transcripts in their GAS muscles**. Representative RT-PCR analysis showing the splicing patterns *Atp2a1* exon22, *Bin1* exon11, *Clcn1* exon7a and *Ryr1* exon70 in the GAS of BMC-25- and AICAR-treated 3–7 -month-old female (A) and male (F) DM1 (HSA^LR^) mice. Percentages of *Atp2a1* exon22 inclusion (B, G), *Bin1* exon11 inclusion (C, H), *Clcn1* exon7a exclusion (D, I), and *Ryr1* exon70 inclusion (E, J) are shown with mean ± SEM in female (B–E) and male (G–J) DM1 mice. Wild-type (WT) data is represented by the horizontal line on the graphs in panels B-E and G-J. *N* = 3–6. AICAR: 500 mg/kg of AICAR, positive control, BMC-25: 1.67 mg/kg of Bitter melon compound. (A Welch’s one-tail *t*-test was performed ∗*P*-value <0.05, ∗∗*P*-value ≤0.01, ∗∗∗*P*-value ≤0.001 and ∗∗∗∗P- value ≤ 0.0001 relative to the vehicle).

Our investigation of the effect of BMC-25 treatment on splicing in TA muscles of DM1 mice indicated a more pronounced therapeutic effect in female DM1 mice compared to their male counterparts. To further investigate these sex-dependent differences, we conducted additional analyses using the gastrocnemius (GAS) muscle. Consistent with previous work, long-term treatment of AICAR in female DM1 mice showed a significant improvement of aberrantly spliced transcripts (Fig. 8A–E), with a more improved phenotype in the female treated DM1 mice in comparison to their male counterparts (Fig. 8F–J) [39,40]. Treatment with BMC-25 in the female DM1 cohort significantly (*P* < 0.05 for *Atp2a1* exon 22, *P* < 0.01 for *Bin1* exon 11, *P* < 0.05 for *Clcn1* exon 7a, and *P* < 0.01 for *Ryr1* exon 70) corrected all alternative splicing events (ASE) in the GAS muscle (Fig. 8A–E) with no significant correction of splicing events in BMC-25-treated male DM1 mice (Fig. 8F–J).

Taken together, these, results indicate that long-term treatment of DM1 mice with BMC-25 results in the correction of splicing events in a sex-specific manner.

## Discussion

The objectives of this study were to characterize novel natural compounds that activate AMPK as a therapeutic strategy for DM1. Through a screen of BMCs, we identified BMC-25 as a promising compound that activates AMPK in DM1 skeletal muscle, thereby highlighting its potential as a therapeutic for DM1 (Fig. 1, Fig. 2). We further show that long-term treatment of DM1 mice with BMC-25 results in the reduction of central nucleation and hypertrophic fibers while also decreasing the presence of RNA foci (Fig. 3, Fig. 4, Fig. 5). Moreover, our findings also demonstrate that long-term treatment of BMC-25 normalized CELF1, MBNL1 and Staufen1 levels in a sex-specific manner with only females showing improvements (Fig. 6, Table 3). Finally, BMC-25 rescued the aberrant splicing patterns of several transcripts in female DM1 mice but not in the male cohort (Fig. 7, Fig. 8, Table 3). Taken together, these results show that BMC-25 improves the histopathological muscle phenotype of both male and female DM1 mice but provides marked molecular benefits only to female DM1 mice.

**Table 3:** Impact of chronic BMC-25 treatment on DM1 pathological features in HSA^LR^ mice.

DM1 pathological features	Female DM1 mice	Male DM1 mice
Rectification of CELF1 RBP levels	Yes	No
Alteration in MBNL1 RBP levels	Yes	Yes
Alteration in Staufen1 RBP levels	Strong trend toward reduction	No
Correction of *ATP2a1* transcript levels- TA	Yes	Strong trend toward correction
Correction of *Bin1* transcript levels- TA	Yes	No
Correction of *Cln1* transcript levels- TA	No	No
Correction of *Ryr1* transcript levels- TA	No	No
Correction of *ATP2a1* transcript levels-GAS	Yes	No
Correction of *Bin1* transcript levels-GAS	Yes	No
Correction of *Cln1* transcript levels-GAS	Yes	No
Correction of *Ryr1* transcript levels-GAS	Yes	No

### Novel natural compounds to improve the DM1 skeletal muscle phenotype

As of date, therapeutic interventions for DM1 patients consist of symptomatic relief through antidiabetic, anti-myotonic, analgesic agents and rehabilitative treatment for strengthening muscles and maximizing patient independence [73,74]. While these traditional therapies improve the patient’s quality of life, they lack true disease-modifying capabilities. As a result, current experimental strategies are shifting toward molecular interventions, including (i) antisense oligonucleotides (AON) aimed at preventing toxic RNA foci, (ii) targeting *DMPK* mRNAs, (iii) the inhibition of MBNL1 sequestration by AON or small molecules, (iv) rescuing RNA-binding proteins levels, and (v) correcting aberrant pre-mRNA splicing via AON and exon skipping strategies [[75], [76], [77]]. Although these therapies remain very promising, they may exhibit challenges in delivery efficiency, tissue specificity, viral-based immunogenic responses, and extreme pricing challenges for DM1 patients.

In attempt to address some of these limitations, recent studies have focused on modulating dysregulated signaling pathways as a therapeutic strategy for DM1 [16]. Inhibitors of the PKC, GSK-3 β, and Akt-mTOR pathways ameliorates CELF1-splicing defects, improves cardiac symptoms, and skeletal muscle histology, including central nucleation, myotonia, muscle atrophy, and splicing defects [16]. More specifically, a current phase II clinical trial with Tideglusib, targeting the GSK-3 β pathway, has shown promise in targeting aberrant molecular and behavioral DM1 phenotypes [78].

Another promising therapeutic avenue for DM1 is the activation of the AMPK pathway through physiological (exercise) or pharmacological means [16]. Our present results show that BMC-25 is a promising clinical candidate due to its plant-derived origin, high bioavailability, and established safety profile. Furthermore, its potency at 1.67 mg/kg makes it ideal for combinatorial regimens. Future research should optimize dose-escalation studies, different treatment regimens, frequency and routes of administration to further rectify signaling defects as well as histological, molecular and functional parameters in both male and female DM1 cohorts.

Other studies have also explored the potential of natural compounds for DM1, including several molecules reported to activate AMPK. While the natural compound resveratrol restores splicing across DM1 models [42,43], metformin’s clinical benefits are limited to improving mobility and hyperglycemia, with no effects observed on myotonia and muscle strength [41]. Furthermore, small molecules like Harmine, Quercetin, and Boldine show varying degrees of efficacy in DM1 models, and indirectly activate AMPK in preclinical studies [77,[79], [80], [81]]. Indeed, Harmine and the flavonoid Quercetin demonstrated robust therapeutic potential by correcting key missplicing events, while Quercetin also reduced *DMPK* expression levels and toxic RNA [80]. In contrast, Boldine appears to operate through a more restricted mechanism where it only reduced RNA aggregates and improved myotonia. While these natural compounds, including BMC-25, demonstrate the ability to target key aspects of the DM1 pathomechanism [77,[79], [80], [81]], they may lack sufficient therapeutic efficacy as standalone therapeutics. Therefore, it appears worthwhile to envisage using a combinatorial approach which multiple compounds together with genetic-based interventions to enhance the efficacy of existing strategies for DM1.

### Sex-specific effects of BMC-25 in the context of the DM1 pathophysiology

DM1 manifests as a clinically heterogenous disorder with significant sex-specific disparities that are often overlooked in standard treatment. Emerging evidence indicates that biological sex heavily influences the disease progression. In particular, female patients typically experience more multisystemic complications involving the ophthalmological, gastrointestinal, and endocrine systems while male patients experience higher morbidity and are more prone to severe skeletal muscular, cardiac, and respiratory impairments [[82], [83], [84]]. These findings suggest that hormonal and metabolic differences may influence the underlying pathology which highlights the necessity of evaluating novel therapeutic interventions such as natural compounds, within the context of this biological variability.

Histological assessment of DM1 mice following long-term BMC-25 treatment shows similar results across both sexes in several key parameters, including central nucleation, cross-sectional area, and toxic RNA aggregates. In contrast, our current molecular data reveal a clear sex-specific response to AMPK activation (Fig. 6, Fig. 7, Fig. 8, Table 3), which is consistent with our previous work. Indeed, our previous findings showed that while MK-8722 (a direct allosteric AMPK activator) reduced central nucleation and hypertrophic fibers in both sexes, it only corrected *Atp2a1* missplicing in males. In contrast, AICAR (a pan-AMPK activator) corrected splicing in both sexes, suggesting that pan-activation, which may induce more widespread metabolic effects, offers a more pronounced therapeutic effect than the direct allosteric activation by MK-8722 [85]. This is further supported by the additive impact between exercise and AICAR, which significantly improved the muscle histology of both sexes, but improved the splicing aberrations particularly in female DM1 mice. This suggests that the female cohort may derive additional biological advantages from this combinatorial therapy. Along those lines, Carrascosa-Saez et al. (2025) [86] recently characterized HSA^LR^ DM1 mice by assessing potential sex-specific differences on several functional, histological, molecular and biochemical parameters. They found no baseline sex differences in histopathology or key splicing events (e.g., *Atp2a1*, *Bin1*), despite variations in plasma biochemical profiles which are in accordance with differences seen in DM1 patients. Nonetheless, these baseline similarities do not preclude sex-specific therapeutic outcomes. The differences in molecular restoration observed here in BMC-25-treated females, which is absent in males, indicates that the female cohort is uniquely responsive to this treatment and can achieve molecular benefits that extend beyond general histological repair.

The molecular and histological improvements seen following BMC-25 treatment are expected to translate into benefits for muscle physiology and function. For example, misregulated RBPs and the resulting missplicing of targets such as *Atp2a1* and *Bin1* cause impairments in muscle relaxation together with muscle structural weakness. Through the correction of these RBPs and the spliceopathy, albeit in a sex-specific manner, and improvements in overall muscle histology in both sexes, one can reasonably assume that BMC-25 treatment would lead to measurable improvements in muscle function, including force generation, motor coordination as well as improvements in the neuromuscular junction biology. Such positive functional adaptations are indeed likely to develop following treatment with BMC-25 given the role of AMPK in the maintenance, remodeling and plasticity of the neuromuscular system [99], and the recent demonstration that wild bitter melon reduces inflammation, oxidative stress, muscle damage and muscle atrophy in male BALB/c mice [100].

The molecular analyses of RBP levels and misspliced transcripts following BMC-25 treatment revealed a distinct sex-dependent molecular response (Fig. 6, Table 3). Indeed, in female DM1 mice, BMC-25 treatment significantly improved the expression levels of CELF1, MBNL1 and Staufen1 while also restoring in parallel, alternative splicing of various transcripts in comparison to their male counterparts. While CELF1 and MBNL1 are primary drivers in the DM1 pathogenesis, Staufen1 also regulates splicing as well as many other aspects of mRNA metabolism including stability and localization [87]. These RBPs function by cooperating to govern the metabolic fate of mRNAs [[87], [88], [89], [90], [91], [92]]. As demonstrated by Wang et al. (2015), hundreds of mRNAs are bound by both MBNL1 and CELF1 within their 3′UTRs [92]. The localization, half-life and alternative splicing of these transcripts are dictated by the competing activities of these RBPs and others. Moreover, work from our laboratory suggests that the reduction of CELF1 and MBNL1 likely lowers Staufen1 levels, creating a feedback loop that further stabilizes the cellular environment [87]. In contrast, treated males exhibit a reduction only in MBNL1. This limited RBP correction explains why males show only a trend toward splicing correction, whereas females achieve a comprehensive molecular rescue.

We postulate that the underlying mechanism driving the therapeutic effects of BMC-25 in both male and female DM1 mice involves the activation of the AMPK pathway. Specifically, BMC-25-mediated AMPK activation may modulate key RBPs which in turn, could enhance the degradation of toxic mutant RNAs. This degradation subsequently liberates sequestered MBNL1 which restores its functional capacity for splicing regulation and thus, corrects the downstream targets implicated in the DM1 multisystemic phenotype. In addition to the demonstrated ability of BMC-25 to disassemble RNA foci and modify the availability and sequestration of MBNL1 (Fig. 4, Fig. 5, Fig. 6), BMC-25 may also reduce mutant *Dmpk* expression, alter RNA processing and impact the assembly of toxic RNA foci. Future studies to definitively validate these proposed mechanisms will more thoroughly establish the therapeutic mechanism of BMC-25.

### Putative mechanism for sex-specific difference

**Fig. 9:**
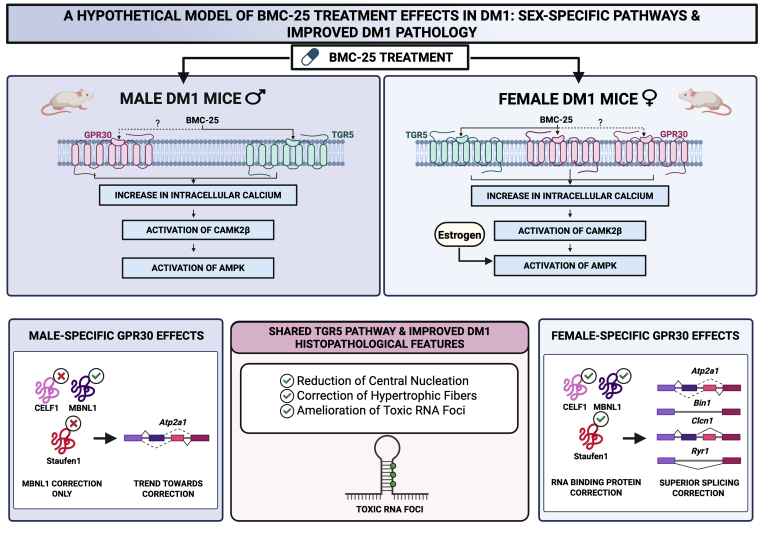
Proposed BMC-25 signaling pathway in male and female DM1 mice. BMC-25 induces TGR5 in both male and female DM1 mice for improved histological pathological characteristics through the CAMK2 β-AMPK axis. Robust induction of GPR30 in female DM1 mice leads to superior RNA splicing correction of multiple transcripts via broad RBP rescue, while mild induction of GPR30 in male DM1 mice shows only MBNL1-mediated trend of Atp2a1. BMC-25 stimulated induction of TGR5 in both sexes in cooperation with GPR30 leads to improved molecular and histopathological features. Dotted line and question mark represents the inferred GPR30 pathway in which BMC-25 exhibits its sex-dependent differences. Figure created by Biorender

**Fig. S1:**
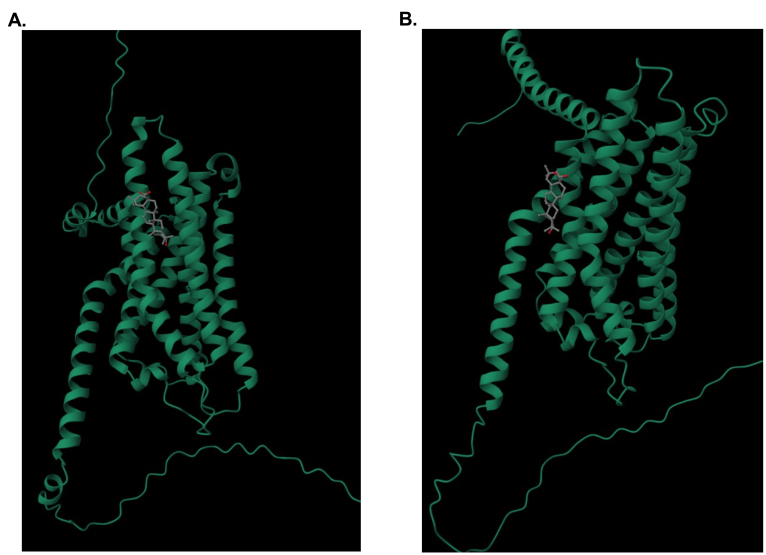
S1. Predicted Binding of BMC-25 to GPR30 Transmembrane Domains. *In silico* docking of BMC-25 (gray) to GPR30 (green). Molecular docking was performed using DiffDock-L on the Neurosnap platform to evaluate the binding affinity of BMC-25 across two distinct conformations of the GPR30 receptor. Poses were selected based on the lowest SMINA Minimized Affinity (highest binding stability). (A) The top-ranked pose for the first receptor conformation demonstrates a SMINA Minimized Affinity of −7.28 kcal/mol. (B) The top-ranked pose for the second receptor conformation exhibits a nearly identical SMINA Minimized Affinity of −7.27 kcal/mol. In both receptor conformations, BMC-25 consistently docked within the transmembrane bundle (TM), specifically localized near the mid-region of the alpha-helical structures. The TM area is typically where GPCRs bind their ligands. The convergence of these high-affinity poses across different conformations suggests a robust and specific binding pocket for BMC-25 within GPR30, supporting its potential role as a direct modulator of the receptor.

The observation of sex-specific responses to BMC-25 likely stems from differences in pharmacokinetics and dimorphic receptor density profiles. Beyond drug metabolism, underlying variations in hormonal modulation may render specific pathways more susceptible to pharmacological intervention and therefore, differential therapeutic outcomes. Thus, we present here a working hypothetical model to outline a potential mechanism for the observed sex-specific effects, putatively involving the receptors GPR30 and TGR5 (Fig. 9). BMC-25 is known to act via the bile acid GPCR, TGR5, which regulates energy metabolism and muscle health by stimulating the AKT/mTOR pathway and MyoG transcription [93]. TGR5 also functions by suppressing atrophy-related genes like *atrogin-1* [93]. This TGR5-AMPK axis likely facilitates the histological improvements observed in both sexes. However, TGR5 activation alone does not explain the superior splicing and CELF1, MBNL1 and Staufen1 modulations seen specifically in females. We therefore speculate that BMC-25 exerts its effects via membrane estrogen GPCRs [67,94], specifically GPR30. Indeed, this receptor is not only expressed at significantly higher levels in female skeletal muscle, but is also known to be targeted by certain BMCs which act as estrogen receptor agonists with potent metabolic benefits [56,67,95]. *In silico* ligand docking performed with the DiffDock-L algorithm on the Neurosnap platform supports this hypothesis, whereby it identified potential binding sites for BMC-25 on GPR30 with strong predicted binding energies (Supplementary Fig. 1). Ultimately, the simultaneous activation of GPR30 and TGR5 suggests a multi-targeted approach that not only corrects splicing but also fosters a metabolic and structural environment conducive to muscle repair and stability. The convergence of these pathways, particularly the more robust activation of GPR30 in females, likely results in the superior therapeutic benefits observed in the female cohort (Fig. 9). We emphasize that this activation cascade, involving two putative receptors in male and female DM1 mouse muscles, remains hypothetical at this stage. Further investigation into these integrated signaling dynamics thus appears warranted.

This sexual dimorphism provides a robust signaling scaffold, allowing BMC-25 to act as a dual agonist for GPR30 and TGR5. This dual activation triggers the CAMK2β-AMPK signaling axis [67] which corrects RBP missplicing and RBP expression profiles (Fig. 9). In contrast, the lower density of GPR30 and estrogen levels in male DM1 mice likely limits the therapeutic response to TGR5-mediated histological stability, leaving molecular defects largely unaddressed. Additionally, estrogen has also been shown to induce AMPK activation [96], which may cooperate with BMC-25 and relay a multitude of beneficial downstream effects in female muscle. These observations also emphasize that BMC-25 functions through distinct intracellular cascades that are distinct in males versus females. Altogether, these observations highlight the necessity of incorporating sex-specific paradigms into preclinical investigations of DM1 therapeutics.

From a clinical perspective and in a broader disease context, the overlapping signaling dysregulation, histopathological defects, and metabolic disorders shared in DM1 are also implicated in other neuromuscular diseases, namely in Duchenne Muscular Dystrophy (DMD). Indeed, our lab and others have shown that AMPK activation also results in several beneficial adaptations in skeletal muscle of DMD mice [97,98]. It appears therefore warranted to extend the scope of BMC-25 from DM1 to other related disorders through continued investigations to determine its receptor-level interactions, bioavailability in skeletal muscle, and long-term safety profiles *in vivo*. In conclusion, BMC-25 offers a novel therapeutic avenue for treating the DM1 muscle pathology and supports the broader exploration of natural compounds for neuromuscular disease intervention.

## Author contributions

B.J.J. conceived the study with input from A.R.C. and Y.Y..S.A.A., A.R.C., and B.J.J. designed the experiments. Experimental procedures were primarily carried out by S.A.A. and supported by C.F.. J.L., C.T. and Y.Y. provided key reagents and insight into the molecular structure of BMC. Data analyses were conducted by S.A.A., A.R.C., and B.J.J.. The manuscript was written by S.A.A. and B.J.J., and refined through the collective efforts of S.A.A., C.F., J.L., C.T., Y.Y., A.R.C., and B.J.J..

## Declaration of competing interest

The authors declare that they have no known competing financial interests or personal relationships that could have appeared to influence the work reported in this paper.

## References

[1] Turner C, Hilton-Jones D. (2010). The myotonic dystrophies: diagnosis and management. J Neurol Neurosurg Psychiatry.

[2] Yotova V, Labuda D, Zietkiewicz E, Gehl D, Lovell A, Lefebvre JF (2005). Anatomy of a founder effect: myotonic dystrophy in Northeastern Quebec. Hum Genet.

[3] Brook JD, McCurrach ME, Harley HG, Buckler AJ, Church D, Aburatani H (1992). Molecular basis of myotonic dystrophy: expansion of a trinucleotide (CTG) repeat at the 3’ end of a transcript encoding a protein kinase family member. Cell.

[4] Fu YH, Pizzuti A, Fenwick RG, King J, Rajnarayan S, Dunne PW (1992). An unstable triplet repeat in a gene related to myotonic muscular dystrophy. Science.

[5] Michalowski S, Miller JW, Urbinati CR, Paliouras M, Swanson MS, Griffith J. (1999). Visualization of double-stranded RNAs from the myotonic dystrophy protein kinase gene and interactions with CUG-binding protein. Nucleic Acids Res.

[6] Mankodi A, Logigian E, Callahan L, McClain C, White R, Henderson D (2000). Myotonic dystrophy in transgenic mice expressing an expanded CUG repeat. Science.

[7] Thornton C.A. (2014). Myotonic dystrophy. Neurol Clin.

[8] Bird TD. Myotonic Dystrophy Type 1. 1999 Sep 17 [updated 2024 Nov 14]. In: Adam MP, Bick S, Mirzaa GM, Pagon RA, Wallace SE, et al., editors. GeneReviews® [Internet]. Seattle (WA): University of Washington, Seattle; 1993–2026. Available from: https://www.ncbi.nlm.nih.gov/books/NBK1165/

[9] Liao Q, Zhang Y, He J, Huang K. (2022). Global prevalence of myotonic dystrophy: an updated systematic review and meta-analysis. Neuroepidemiology.

[10] Mahadevan M, Tsilfidis C, Sabourin L, Shutler G, Amemiya C, Jansen G (1992). Myotonic dystrophy mutation: an unstable CTG repeat in the 3′ untranslated region of the gene. Science.

[11] Taneja KL, McCurrach M, Schalling M, Housman D, Singer RH. (1995). Foci of trinucleotide repeat transcripts in nuclei of myotonic dystrophy cells and tissues. J Cell Biol.

[12] Davis BM, McCurrach ME, Taneja KL, Singer RH, Housman DE. (1997). Expansion of a CUG trinucleotide repeat in the 3′ untranslated region of myotonic dystrophy protein kinase transcripts results in nuclear retention of transcripts. Proc Natl Acad Sci USA.

[13] Miller JW, Urbinati CR, Teng-Umnuay P, Stenberg MG, Byrne BJ, Thornton CA (2000). Recruitment of human muscleblind proteins to (CUG)(n) expansions associated with myotonic dystrophy. EMBO J.

[14] López-Martínez A, Soblechero-Martín P, de-la-Puente-Ovejero L, Nogales-Gadea G, Arechavala-Gomeza V. (2020). An overview of alternative splicing defects implicated in myotonic dystrophy type I. Genes (Basel).

[15] Ho TH, Charlet B N, Poulos MG, Singh G, Swanson MS, Cooper TA. (2004). Muscleblind proteins regulate alternative splicing. EMBO J.

[16] Ravel-Chapuis A, Atieh SA, Fahmi C, Jasmin BJ. (2026). Disruptions of cell signaling pathways in myotonic dystrophy type 1 (DM1) skeletal muscle, their pathogenic impact and potential for combinatorial therapeutics. J Biol Chem.

[17] Tian B, White RJ, Xia T, Welle S, Turner DH, Mathews MB (2000). Expanded CUG repeat RNAs form hairpins that activate the double-stranded RNA-dependent protein kinase PKR. RNA.

[18] Jiang H, Mankodi A, Swanson MS, Moxley RT, Thornton CA. (2004). Myotonic dystrophy type 1 is associated with nuclear foci of mutant RNA, sequestration of muscleblind proteins and deregulated alternative splicing in neurons. Hum Mol Genet.

[19] Kuyumcu-Martinez NM., Wang GS., Cooper TA. (2007). Increased steady-state levels of CUGBP1 in myotonic dystrophy 1 are due to PKC-mediated hyperphosphorylation. Mol Cell.

[20] Jones K, Wei C, Iakova P, Bugiardini E, Schneider-Gold C, Meola G (2012). GSK3β mediates muscle pathology in myotonic dystrophy. J Clin Invest.

[21] Yadava RS, Foff EP, Yu Q, Gladman JT, Kim YK, Bhatt KS (2015). TWEAK/Fn14, a pathway and novel therapeutic target in myotonic dystrophy. Hum Mol Genet.

[22] Brockhoff M, Rion N, Chojnowska K, Wiktorowicz T, Eickhorst C, Erne B (2017). Targeting deregulated AMPK/mTORC1 pathways improves muscle function in myotonic dystrophy type I. J Clin Invest.

[23] Song KY, Guo XM, Wang HQ, Zhang L, Huang SY, Huo YC (2020). MBNL1 reverses the proliferation defect of skeletal muscle satellite cells in myotonic dystrophy type 1 by inhibiting autophagy via the mTOR pathway. Cell Death Dis.

[24] Ravel-Chapuis A, Bélanger G, Côté J, Michel RN, Jasmin BJ. (2017). Misregulation of calcium-handling proteins promotes hyperactivation of calcineurin–NFAT signaling in skeletal muscle of DM1 mice. Hum Mol Genet.

[25] Ravel-Chapuis A, Al-Rewashdy A, Bélanger G, Jasmin BJ. (2018). Pharmacological and physiological activation of AMPK improves the spliceopathy in DM1 mouse muscles. Hum Mol Genet.

[26] Dabo S, Meurs EF. (2012). dsRNA-Dependent protein kinase PKR and its role in stress, signaling and HCV infection. Viruses.

[27] Schmitz-Peiffer C, Biden TJ. (2008). Protein kinase C function in muscle, liver, and β-Cells and its therapeutic implications for type 2 diabetes. Diabetes.

[28] Duquesnes N, Lezoualc’h F, Crozatier B. (2011). PKC-delta and PKC-epsilon: foes of the same family or strangers?. J Mol Cell Cardiol.

[29] Beurel E, Grieco SF, Jope RS. (2015). Glycogen synthase kinase-3 (GSK3): regulation, actions, and diseases. Pharmacol Ther.

[30] Panwar V, Singh A, Bhatt M, Tonk RK, Azizov S, Raza AS (2023). Multifaceted role of mTOR (mammalian target of rapamycin) signaling pathway in human health and disease. Signal Transduct Target Ther.

[31] Bodine SC. (2022). The role of mTORC1 in the regulation of skeletal muscle mass. Fac Rev.

[32] Laplante M, Sabatini DM. (2012). mTOR signaling in growth control and disease. Cell.

[33] Mounier R, Théret M, Lantier L, Foretz M, Viollet B. (2015). Expanding roles for AMPK in skeletal muscle plasticity. Trends Endocrinol Metab.

[34] Sato S, Ogura Y, Kumar A. (2014). TWEAK/Fn14 signaling axis mediates skeletal muscle atrophy and metabolic dysfunction. Front Immunol.

[35] Mourkioti F, Rosenthal N. (2008). NF-κB signaling in skeletal muscle: prospects for intervention in muscle diseases. J Mol Med (Berl).

[36] Hudson MB., Price SR. (2013). Calcineurin: a poorly understood regulator of muscle mass. Int J Biochem Cell Biol.

[37] Tavi P, Westerblad H. (2011). The role of *in vivo* Ca2+ signals acting on Ca2+–calmodulin-dependent proteins for skeletal muscle plasticity. J Physiol.

[38] Chin ER, Olson EN, Richardson JA, Yang Q, Humphries C, Shelton JM (1998). A calcineurin-dependent transcriptional pathway controls skeletal muscle fiber type. Genes Dev.

[39] Misquitta NS., Ravel-Chapuis A, Jasmin BJ. (2023). Combinatorial treatment with exercise and AICAR potentiates the rescue of myotonic dystrophy type 1 mouse muscles in a sex-specific manner. Hum Mol Genet.

[40] Ravel-Chapuis A, Fahmi C, Gobin J, Jasmin BJ. (2024). The AMPK allosteric activator MK-8722 improves the histology and spliceopathy in myotonic dystrophy type 1 (DM1) skeletal muscle. FASEB J.

[41] Bassez G, Audureau E, Hogrel JY, Arrouasse R, Baghdoyan S, Bhugaloo H (2018). Improved mobility with metformin in patients with myotonic dystrophy type 1: a randomized controlled trial. Brain.

[42] Santoro M, Piacentini R, Perna A, Pisano E, Severino A, Modoni A (2020). Resveratrol corrects aberrant splicing of RYR1 pre-mRNA and Ca2+ signal in myotonic dystrophy type 1 myotubes. Neural Regen Res.

[43] Takarada T, Nishida A, Takeuchi A, Lee T, Takeshima Y, Matsuo M. (2015). Resveratrol enhances splicing of insulin receptor exon 11 in myotonic dystrophy type 1 fibroblasts. Brain Dev.

[44] Tran N, Pham B, Le L. (2020). Bioactive compounds in anti-diabetic plants: from herbal medicine to modern drug discovery. Biology.

[45] Sridhar MG, Vinayagamoorthi R, Arul Suyambunathan V, Bobby Z, Selvaraj N. (2008). Bitter gourd (Momordica charantia) improves insulin sensitivity by increasing skeletal muscle insulin-stimulated IRS-1 tyrosine phosphorylation in high-fat-fed rats. Br J Nutr.

[46] Shimada T, Kato F, Dwijayanti DR, Nagata T, Kinoshita A, Okuyama T (2022). Bitter melon fruit extract enhances intracellular ATP production and insulin secretion from rat pancreatic β-cells. Br J Nutr.

[47] Ahmed I, Adeghate E, Sharma AK, Pallot DJ, Singh J. (1998). Effects of Momordica charantia fruit juice on islet morphology in the pancreas of the streptozotocin-diabetic rat. Diabetes Res Clin Pract.

[48] Welihinda J, Karunanayake EH, Sheriff MH, Jayasinghe KS. (1986). Effect of Momordica charantia on the glucose tolerance in maturity onset diabetes. J Ethnopharmacol.

[49] Miura T, Itoh C, Iwamoto N, Kato M, Kawai M, Park SR (2001). Hypoglycemic activity of the fruit of the Momordica charantia in type 2 diabetic mice. J Nutr Sci Vitaminol.

[50] Sarkar S, Pranava M, Marita R. (1996). Demonstration of the hypoglycemic action of *Momordica charantia* in a validated animal model of diabetes. Pharmacol Res.

[51] Joseph B, Jini D. (2013). Antidiabetic effects of Momordica charantia (bitter melon) and its medicinal potency. Asian Pac J Trop Dis.

[52] Shih CC., Lin CH, Lin WL. (2008). Effects of Momordica charantia on insulin resistance and visceral obesity in mice on high-fat diet. Diabetes Res Clin Pract.

[53] Bora AFM, Kouame KJEP, Li X, Liu L, Pan Y. (2023). New insights into the bioactive polysaccharides, proteins, and triterpenoids isolated from bitter melon (Momordica charantia) and their relevance for nutraceutical and food application: a review. Int J Biol Macromol.

[54] Mukherjee S, Karati D. (2023). Exploring the phytochemistry, pharmacognostic properties, and pharmacological activities of medically important plant *momordica charantia*. Pharmacol Res - Modern Chin Med.

[55] Sun K, Ding M, Fu C, Li P, Li T, Fang L (2023). Effects of dietary wild bitter melon (Momordica charantia var. abbreviate Ser.) extract on glucose and lipid metabolism in HFD/STZ-induced type 2 diabetic rats. J Ethnopharmacol.

[56] Tan MJ, Ye JM, Turner N, Hohnen-Behrens C, Ke CQ, Tang CP (2008). Antidiabetic activities of triterpenoids isolated from bitter melon associated with activation of the AMPK pathway. Chem Biol.

[57] Bhattacharya S, Muhammad N, Steele R, Kornbluth J, Ray RB. (2017). Bitter Melon enhances natural killer–mediated toxicity against head and neck cancer cells. Cancer Prev Res.

[58] Bhattacharya S, Muhammad N, Steele R, Peng G, Ray RB. (2016). Immunomodulatory role of bitter melon extract in inhibition of head and neck squamous cell carcinoma growth. Oncotarget.

[59] Jiang Y, Miao J, Wang D, Zhou J, Liu B, Jiao F (2018). MAP30 promotes apoptosis of U251 and U87 cells by suppressing the LGR5 and Wnt/β-catenin signaling pathway, and enhancing Smac expression. Oncol Lett.

[60] Kwatra D, Subramaniam D, Ramamoorthy P, Standing D, Moran E, Velayutham R (2013). Methanolic extracts of bitter melon inhibit colon cancer stem cells by affecting energy homeostasis and autophagy. Evid Based Complement Alternat Med.

[61] Fang EF, Froetscher L, Scheibye-Knudsen M, Bohr VA, Wong JH, Ng TB. Emerging Antitumor Activities of the Bitter Melon (Momordica charantia). Curr Protein Pept Sci. 2019;20(3):296–301. doi:10.2174/1389203719666180622095800 PubMed PMID: 29932035; PubMed Central PMCID: PMC6486373. 10.2174/1389203719666180622095800 PMC6486373 29932035

[62] Sur S, Nakanishi H, Flaveny C, Ippolito JE, McHowat J, Ford DA (2019). Inhibition of the key metabolic pathways, glycolysis and lipogenesis, of oral cancer by bitter melon extract. Cell Commun Signal.

[63] Thiagarajan S, Arapoc DJ, Shafie NH, Keong YY, Bahari H, Adam Z (2019). Momordica charantia (Indian and Chinese bitter Melon) extracts inducing apoptosis in human lung cancer cell line A549 via ROS-mediated mitochodria injury. Evid Based Complement Alternat Med.

[64] Richter E, Geetha T, Burnett D, Broderick TL, Babu JR. (2023). The effects of Momordica charantia on type 2 diabetes mellitus and Alzheimer’s disease. Int J Mol Sci.

[65] Gao Y, Li X, Huang Y, Chen J, Liu Y, Qiu M. (2023). Bitter melon and diabetes mellitus. Food Rev Int.

[66] Ravel-Chapuis A, Duchesne E, Jasmin BJ. (2022). Pharmacological and exercise-induced activation of AMPK as emerging therapies for myotonic dystrophy type 1 patients. J Physiol.

[67] Iseli TJ, Turner N, Zeng XY, Cooney GJ, Kraegen EW, Yao S (2013). Activation of AMPK by bitter melon triterpenoids involves CaMKKβ. PLoS One.

[68] André LM, Ausems CRM, Wansink DG, Wieringa B. (2018). Abnormalities in skeletal muscle myogenesis, growth, and regeneration in myotonic dystrophy. Front Neurol.

[69] Lin X, Miller JW, Mankodi A, Kanadia RN, Yuan Y, Moxley RT (2006). Failure of MBNL1-dependent post-natal splicing transitions in myotonic dystrophy. Hum Mol Genet.

[70] Fugier C, Klein AF, Hammer C, Vassilopoulos S, Ivarsson Y, Toussaint A (2011). Misregulated alternative splicing of BIN1 is associated with T tubule alterations and muscle weakness in myotonic dystrophy. Nat Med.

[71] Lee KY, Li M, Manchanda M, Batra R, Charizanis K, Mohan A (2013). Compound loss of muscleblind-like function in myotonic dystrophy. EMBO Mol Med.

[72] Kino Y, Washizu C, Oma Y, Onishi H, Nezu Y, Sasagawa N (2009). MBNL and CELF proteins regulate alternative splicing of the skeletal muscle chloride channel CLCN1. Nucleic Acids Res.

[73] (2026). What DM treatment or therapies are available?.

[74] Dystrophy Myotonic (2026). https://my.clevelandclinic.org/health/diseases/24516-myotonic-dystrophy-dm.

[75] Magaña JJ, Cisneros B. (2011). Perspectives on gene therapy in myotonic dystrophy type 1. J Neurosci Res.

[76] Overby SJ, Cerro-Herreros E, Llamusi B, Artero R (2018). RNA-mediated therapies in myotonic dystrophy. Drug Discov Today.

[77] López-Morató M, Brook JD, Wojciechowska M. (2018). Small molecules which improve pathogenesis of myotonic dystrophy type 1. Front Neurol.

[78] Horrigan J, Gomes TB, Snape M, Nikolenko N, McMorn A, Evans S (2020). A phase 2 study of AMO-02 (tideglusib) in congenital and childhood-onset myotonic dystrophy type 1 (DM1). Pediatr Neurol.

[79] Herrendorff R, Faleschini MT, Stiefvater A, Erne B, Wiktorowicz T, Kern F (2016). Identification of plant-derived alkaloids with therapeutic potential for myotonic dystrophy type I. J Biol Chem.

[80] Mishra SK, Hicks SM, Frias JA, Vangaveti S, Nakamori M, Cleary JD, et al. Quercetin selectively reduces expanded repeat RNA levels in models of myotonic dystrophy. *bioRxiv* [Preprint]. 2023 [cited 2026 Jul 26]:2023.02.02.526846. Available from: https://www.biorxiv.org/content/10.1101/2023.02.02.526846v1

[81] Álvarez-Abril MC, García-Alcover I, Colonques-Bellmunt J, Garijo R, Pérez-Alonso M, Artero R (2023). Natural compound boldine lessens myotonic dystrophy type 1 phenotypes in DM1 drosophila models, patient-derived cell lines, and HSALR mice. Int J Mol Sci.

[82] Chakraborty D, Borthakur S, Sarkar R (2025). Singh MD.Gender disparities in myotonic dystrophy 1. Life Sci.

[83] Dogan C, De Antonio M, Hamroun D, Varet H, Fabbro M, Rougier F (2016). Gender as a modifying factor influencing myotonic dystrophy type 1 phenotype severity and mortality: a nationwide multiple databases cross-sectional observational study. PLoS One.

[84] Perna A, Maccora D, Rossi S, Nicoletti TF, Zocco MA, Riso V (2020). High prevalence and gender-related differences of gastrointestinal manifestations in a cohort of DM1 patients: a perspective, cross-sectional study. Front Neurol.

[85] Višnjić D, Lalić H, Dembitz V, Tomić B, Smoljo T (2021). AICAr, a widely used AMPK activator with important AMPK-independent effects: a systematic review. Cells.

[86] Carrascosa-Sàez M, Colom-Rodrigo A, González-Martínez I, Pérez-Gómez R, García-Rey A, Piqueras-Losilla D (2025). Use of HSALR female mice as a model for the study of myotonic dystrophy type I. Lab Anim.

[87] Crawford Parks TE, Marcellus KA, Péladeau C, Jasmin BJ, Ravel-Chapuis A. (2020). Overexpression of Staufen1 in DM1 mouse skeletal muscle exacerbates dystrophic and atrophic features. Hum Mol Genet.

[88] David G, Reboutier D, Deschamps S, Méreau A, Taylor W, Padilla-Parra S, et al. (2022). The RNA-binding proteins CELF1 and ELAVL1 cooperatively control the alternative splicing of CD44. Biochem Biophys Res Commun.

[89] David G, Deschamps S, Padilla-Parra S, Tramier M, Audic Y, Paillard L. The RNA-binding proteins CELF1 and ELAVL1 cooperatively control alternative splicing. bioRxiv [Preprint]. 2018 [cited 2026 Mar 30]:373704. Available from: https://www.biorxiv.org/content/10.1101/373704v1. 10.1016/j.bbrc.2022.07.073 35973378

[90] Wagner SD, Struck AJ, Gupta R, Farnsworth DR, Mahady AE, Eichinger K (2016). Dose-dependent regulation of alternative splicing by MBNL proteins reveals biomarkers for myotonic dystrophy. PLoS Genet.

[91] Du H, Cline MS, Osborne RJ, Tuttle DL, Clark TA, Donohue JP (2010). Aberrant alternative splicing and extracellular matrix gene expression in mouse models of myotonic dystrophy. Nat Struct Mol Biol.

[92] Wang ET, Ward AJ, Cherone JM, Giudice J, Wang TT, Treacy DJ (2015). Antagonistic regulation of mRNA expression and splicing by CELF and MBNL proteins. Genome Res.

[93] Sasaki T, Kuboyama A, Mita M, Murata S, Shimizu M, Inoue J (2018). The exercise-inducible bile acid receptor Tgr5 improves skeletal muscle function in mice. J Biol Chem.

[94] Cignarella A, Boscaro C, Albiero M, Bolego C, Barton M. (2023). Post-transcriptional and epigenetic regulation of estrogen signaling. J Pharmacol Exp Ther.

[95] Hsu C, Hsieh CL, Kuo YH, Huang CJ. (2011). Isolation and identification of cucurbitane-type triterpenoids with partial agonist/antagonist potential for estrogen receptors from Momordica charantia. J Agric Food Chem.

[96] Lipovka Y, Chen H, Vagner J, Price TJ, Tsao TS, Konhilas JP. (2015). Oestrogen receptors interact with the α-catalytic subunit of AMP-activated protein kinase. Biosci Rep.

[97] Ljubicic V, Jasmin BJ. (2013). AMP-activated protein kinase at the nexus of therapeutic skeletal muscle plasticity in Duchenne muscular dystrophy. Trends Mol Med.

[98] Ljubicic V, Khogali S, Renaud JM, Jasmin BJ. (2012). Chronic AMPK stimulation attenuates adaptive signaling in dystrophic skeletal muscle. Am J Physiol Cell Physiol.

[99] Dial AG, Ng SY, Manta A, Ljubicic V. (2018). The role of AMPK in neuromuscular biology and disease. Trends Endocrinol Metabol.

[100] Seifi S, Nazari SE, Avan A, Khalili-Tanha N, Babaei F, Soleimanpour S (2024). The therapeutic potential of Wild Bitter Melon to ameliorate muscle atrophy in a murine model. Avicenna J Phytomed.

